# Supplementary material for: Global fossil fuel reduction pathways under different climate mitigation strategies and ambitions
Source: Nat Commun. 2023 Sep 13;14:5425. doi: 10.1038/s41467-023-41105-z (PMC10499994; doi:10.1038/s41467-023-41105-z)
Supplement: Supplementary file 1 — Supplementary Information [file 41467_2023_41105_MOESM1_ESM.pdf]

SUPPLEMENTARY INFORMATION TO "GLOBAL FOSSIL FUEL REDUCTION PATHWAYS UNDER DIFFERENT CLIMATE MITIGATION STRATEGIES AND AMBITIONS"

SUPPLEMENTARY Discussion

Additional results and discussions for "Characteristics of scenarios with different roles for gas" section

C1 scenarios

Figure 4 shows that the "rebound" C1 scenarios are generally associated with one or more of the following features: (1) much higher fossil fuel use coupled to CCS, including for electricity generation from gas; (2) higher CDR via negative AFOLU emissions and DACCS; (3) lower carbon prices, especially after 2050; (4) higher gas demand in the transportation sector; (5) higher primary energy supply from nuclear; (6) lower capacity additions for electricity generation from solar and wind; and (7) lower capital costs for electricity generation from coal-fired power plants (without CCS) but higher from offshore wind. (For the last two features, we interpret the relevant variables with caution give the relatively limited reporting.) Conversely, the "fast decline" scenarios are typically associated with much less reliance on fossil CCS, higher carbon prices, and sustained increases in renewables integration from 2020 onwards. The "slow decline" scenarios share similar characteristics, but generally have higher CDR via BECCS and less extremely high carbon prices seen in some of the "fast decline" scenarios. The reduction pathways for gas-powered electricity generation without CCS are, however, similar between all three clusters, showing an almost complete phase-out by around 2040.

As shown in Supplementary Figures 15-16, all C1 scenarios generated by the following six models/model families (and their associated scenario projects) are grouped into the "rebound" cluster: GCAM 5.3 (Project study: Ou 2021), GEM-E3 (ENGAGE), IMAGE 3.2 (van Vuuren 2021), MESSAGEix-GLOBIOM 1.1 (ENGAGE), MESSAGEix-GLOBIOM 1.2 (Kikstra 2021), and WITCH (ENGAGE). Scenarios grouped into the "fast decline" and "slow decline" clusters belong to multiple projects, but are predominantly from the REMIND model family.

Compared to other scenario projects, the ENGAGE project[1] has the largest number of scenarios represented in each of the C1-C3 categories (Figure S3). Consequently, we pay special attention as to whether scenarios from this project all show relatively higher or lower gas supply values compared to scenarios from other projects within the AR6 scenario ensemble. In each of the C1-C3 categories, we find that scenarios from the ENGAGE project appear in all three gas clusters. The largest number of ENGAGE scenarios is found in the "rebound" cluster in C1, and in the "fast decline" or "decline" clusters in C2 and C3, respectively.

C2 scenarios

The C2 clusters display similar characteristics to those of the C1. Supplementary Figure 13 shows that the "rebound" C2 scenarios are generally associated with one or more of the following features: (1) much higher fossil fuel use coupled to CCS, especially for electricity generation from gas; (2) higher CDR via negative AFOLU emissions and DACCS; (3) lower carbon prices, especially after 2060; (4) higher hydrogen production from gas ; (5) lower capacity additions for electricity generation from solar and wind; and (g) higher capital costs for electricity generation from

offshore wind. (For the last two features, we interpret the relevant variables with caution give the relatively limited reporting.) Conversely, the "fast decline" scenarios are typically associated with much less reliance on fossil CCS, higher carbon prices, and sustained increases in renewables integration from 2020 onwards. The "slow decline" scenarios share similar characteristics, but generally have higher CDR via BECCS and less extremely high carbon prices seen in some of the "fast decline" scenarios. The reduction pathways for gas-powered electricity generation without CCS are, however, similar between all three clusters, showing an almost complete phase-out by around 2050.

As shown in Supplementary Figures 15-16, all 10 C2 scenarios from the GEM-E3_V2021 model (associated with the ENGAGE project) are grouped into the "rebound" cluster. All C2 scenarios from the COFFEE 1.1 and IMAGE 3.2 models, and 25 out of 43 scenarios from the MESSAGE model family, are found in the "slow decline" cluster. All scenarios from the AIM/CGE 2.2 and POLES ENGAGE models and from the WITCH model family, as well as the majority of scenarios from the REMIND model family, are grouped into the "fast decline" cluster.

C3 scenarios

Supplementary Figure 14 shows that the "decline" C3 scenarios are generally associated with one or more of the following features: (1) lower reliance on fossil CCS; (2) lower CDR via AFOLU and DACCS; (3) higher carbon prices after around 2060; (4) higher primary energy supply from renewables, including sustained increases in renewable electricity capacity additions; and (5) higher capital costs for electricity generation from coal-CCS power plants but lower from offshore wind. (For the last two features, we interpret the relevant variables with caution give the relatively limited reporting.) Compared to the "decline" cluster, the "rebound" scenarios rely on higher fossil CCS and CDR via AFOLU and DACCS, have higher gas demand for buildings, and lower capacity additions from solar (but not wind) for electricity generation. Meanwhile, the "increase" cluster is associated with higher levels of fossil CCS, gas demand for transportation, higher CDR via AFOLU and DACCS, and lower capacity additions from renewables. Both the "rebound" and "increase" scenarios generally see carbon prices plateauing after around 2070. As in the C1 and C2 scenarios, the reduction pathways for gas-powered electricity generation without CCS are similar between all three C3 clusters, showing an almost complete phase-out by around 2050.

As shown in Supplementary Figures 15-16, around half of the scenarios in the "rebound" cluster are from the WITCH model family (19 out of 39). All C3 scenarios from the GEM-E3 and IMAGE 3.2 models, all but one scenario from the GCAM 5.3 model, and the majority of scenarios from the MESSAGE model family are grouped into the "increase" cluster. The majority of scenarios from the REMIND, TIAM, POLES, AIM, and COFFEE model families are grouped into the "decline" cluster, with 77 out of 164 scenarios (47%) in this cluster coming from the REMIND model family.

Additional results and discussion for the "Sensitivity of fossil fuel reduction pathways to different mitigation strategies and uncertainties in CDR potential" section

Here we describe the fossil fuel reduction pathways shown in Figure 5 for the five illustrative mitigation pathways (IMPs) of the AR6 in detail.

The IPCC AR6 assessment of all submitted mitigation scenarios identified five different “illustrative mitigation pathways” (IMPs) that reflect different prominent mitigation strategies for reaching a given temperature outcome. There are three IMPs in the C1 category: “IMP-LD” – strong emphasis on energy demand reductions; “IMP-Ren” – heavy reliance on renewables; “IMP-SP” – mitigation in the context of broader sustainable development. “IMP-Neg”, which relies on extensive CDR in the energy and the industry sectors, is a C2 scenario. “IMP-GS”, which entails a less rapid and gradual strengthening of near-term mitigation actions, is a C3 scenario.

In the C1 category, the IMP-LD scenario meets the 1.5°C threshold with no or limited overshoot through much faster reductions in oil and gas – and much higher carbon prices – than almost all other scenarios (Supplementary Fig. 20). For oil, this is accomplished through rapid and dramatic transitions to shared vehicle fleets, flexible transit systems, and rapid vehicle electrification; as well as reductions in freight volumes due to longer-lasting and more material-efficient goods. For gas, this is accomplished through extensive end-use efficiency improvements (e.g., through building retrofits), plus preferring renewable power to gas with CCS. In fact, the IMP-LD scenario deploys no CCS or BECCS for normative reasons, informed by concern over innovation failure, investment risks, and public opposition (though it does rely extensively on CDR via the AFOLU sector)[5].

By contrast, the IMP-Ren scenario lacks as much focus on shared and flexible transit or material and end-use efficiency, but goes even harder on electrification, as powered by renewable electricity[6]. Lastly, IMP-SP phases out all fossil fuels almost completely by the end of the century and with relatively limited CDR and CCS reliance, driven in part by the “substantially reduce[d] detrimental effects of outdoor air pollution on public health”, as well as by additional ecosystem and human health constraints that limit certain mitigation options related to bioenergy and land availability[7]. Still, IMP-SP’s focus on availability of and access to modern energy services (e.g., electric and LPG cook stoves) in developing regions causes it to reduce oil and gas demand slightly slower than do scenarios without this explicit focus on improving livelihoods and limiting cost impacts on low-income households.

In the C2 category, the IMP-Neg scenario allows dramatically more room for coal than most scenarios but, over time, less room for gas (Figure 5). The continued use of coal in this scenario is driven by ongoing use in industry (and to a lesser extent for coal-powered electricity generation coupled to CCS), which is enabled by extensive CDR reliance: up to 8 $GtCO_2$ of BECCS, 1-2 $GtCO_2$ of sequestration through the AFOLU sector, and up to 6 $GtCO_2$ by other methods annually in the latter half of the century (Supplementary Fig. 21). This extensive reliance on long-term CDR is typical of C2 scenarios, in which net negative CO_2 emissions are needed to compensate for emissions in the first half of the century and bring down temperatures after the peak[8].

In the C3 category that limits warming to below 2°C, the IMP-GS scenario is generally well within the interquartile range of scenario outcomes, with the exception that it reduces gas by much more in mid-century than most other scenarios (Figure 5). This is largely because this scenario builds much more renewable power (solar and wind) and electricity storage than do most C3 scenarios (Supplementary Fig. 22), which enables it to phase down gas power more rapidly in the near-term. (Over time, gas supply rebounds slightly due to demand in the buildings and industry sectors, with overall emissions primarily being offset by DACCS.)

For our CDR sensitivity analysis, we find that 4 out of 26 C1, 0 out of 15 C2, and 6 out of 62 C3 scenarios that report the relevant variables do not exceed the limits based on Grant et al. The subsets of “model”--“scenario” (and their project study) that meet these criteria are as follows:

C1
REMIND-MAgPIE 2.1-4.2--CEMICS_SSP1-1p5C-minCDR (Strefler 2021b)
REMIND-MAgPIE 2.1-4.2--CEMICS_SSP2-1p5C-minCDR (Strefler 2021b)
REMIND-MAgPIE 2.1-4.2--SusDev_SDP-PkBudg1000 (Soergel 2021)
REMIND-MAgPIE 2.1-4.3--DeepElec_SSP2_ HighRE_Budg900 (Luderer 2021)

C2
None

C3
REMIND-MAgPIE 2.1-4.2--CEMICS_SSP2-2C-minCDR (Strefler 2021b)
REMIND-MAgPIE 2.1-4.2--EN_NPi2020_1200 (ENGAGE)
REMIND-MAgPIE 2.1-4.2--EN_NPi2020_1200f (ENGAGE)
REMIND-MAgPIE 2.1-4.3--DeepElec_SSP2_ HighRE_Budg1100 (Luderer 2021)
REMIND-MAgPIE 2.1-4.3--DeepElec_SSP2_ HighRE_Budg1300 (Luderer 2021)
WITCH 5.0--CO_Bridge (COMMIT)

SUPPLEMENTARY TABLES

Supplementary Table 1. The years during which global coal, oil, and gas supply peak* and the percentage changes in supply relative to 2020 in the C1-C3 pathways shown in Figure 5. Percent changes shown are rounded to the nearest integer. (*Note: Data are available at 5-year intervals; all C2 scenarios exceed the CDR limits of our sensitivity analysis.)

Fossil fuel	Pathway category	Sets of or individual pathways	Peak year	Percent change relative to 2020 (%)			
				2030	2040	2050	2100
Coal	C1	Median of all pathways	2015	-73	-92	-95	-98
		Median of CDR-limited pathways	2015	-84	-99	-99	-100
		IMP-LD	2010	-75	-90	-98	-100
		IMP-Ren	2020	-90	-99	-99	-99
		IMP-SP	2015	-76	-99	-99	-100
	C2	Median of all pathways	2015	-38	-80	-90	-98
		IMP-Neg	2015	-47	-57	-63	-53
	C3	Median of all pathways	2015	-33	-77	-82	-86
		Median of CDR-limited pathways	2015	-48	-93	-99	-99
		IMP-GS	2020	-27	-70	-75	-87
Oil	C1	Median of all pathways	2020	-9	-32	-62	-80
		Median of CDR-limited pathways	2020	-5	-36	-70	-96
		IMP-LD	2015	-47	-75	-90	-100
		IMP-Ren	2025	-3	-47	-77	-88
		IMP-SP	2020	-4	-21	-51	-95
	C2	Median of all pathways	2025	0	-18	-48	-88
		IMP-Neg	2020	-10	-13	-25	-83
	C3	Median of all pathways	2025	4	-10	-33	-73
		Median of CDR-limited pathways	2030	8	-6	-34	-76
		IMP-GS	2030	9	-12	-40	-88
Gas	C1	Median of all pathways	2020	-11	-38	-42	-54
		Median of CDR-limited pathways	2020	-25	-67	-84	-93
		IMP-LD	2020	-47	-75	-85	-99
		IMP-Ren	2020	-33	-58	-78	-98
		IMP-SP	2020	-2	-38	-68	-96
	C2	Median of all pathways	2030	12	-9	-19	-63
		IMP-Neg	2030	17	5	-22	-82
	C3	Median of all pathways	2030	9	-2	-15	-25
		Median of CDR-limited pathways	2025	4	-34	-59	-57
		IMP-GS	2020	-2	-39	-66	-41

Supplementary Table 2. As in Supplementary Table 1 but for primary energy supply from coal, oil, gas without CCS.

Fossil fuel	Pathway category	Sets of or individual pathways	Peak year	Percent change relative to 2020 (%)			
				2030	2040	2050	2100
Coal w/o CCS	C1	**Median of all pathways**	2015	-76	-97	-98	-100
		**Median of CDR-limited pathways**	2015	-84	-99	-99	-100
		**IMP-LD**	*(no fossil CCS used)*				
		**IMP-Ren**	2020	-90	-99	-99	-99
		**IMP-SP**	2015	-76	-99	-99	-100
	C2	**Median of all pathways**	2015	-42	-90	-97	-99
		**IMP-Neg**	2015	-47	-57	-63	-53
	C3	**Median of all pathways**	2015	-34	-82	-93	-99
		**Median of CDR-limited pathways**	2015	-48	-93	-99	-99
		**IMP-GS**	2020	-27	-80	-88	-100
Oil w/o CCS	C1	**Median of all pathways**	2020	-8	-34	-61	-82
		**Median of CDR-limited pathways**	2020	-5	-36	-70	-96
		**IMP-LD**	*(no fossil CCS used)*				
		**IMP-Ren**	2025	-3	-47	-77	-88
		**IMP-SP**	2020	-4	-21	-51	-95
	C2	**Median of all pathways**	2025	0	-18	-49	-88
		**IMP-Neg**	2020	-10	-13	-25	-83
	C3	**Median of all pathways**	2025	4	-11	-33	-73
		**Median of CDR-limited pathways**	2030	8	-6	-34	-76
		**IMP-GS**	2030	9	-12	-40	-88
Gas w/o CCS	C1	**Median of all pathways**	2020	-22	-54	-69	-70
		**Median of CDR-limited pathways**	2020	-25	-69	-87	-97
		**IMP-LD**	*(no fossil CCS used)*				
		**IMP-Ren**	2020	-33	-59	-78	-98
		**IMP-SP**	2020	-3	-39	-69	-96
	C2	**Median of all pathways**	2030	6	-28	-51	-72
		**IMP-Neg**	2030	17	5	-22	-82
	C3	**Median of all pathways**	2030	7	-18	-38	-57
		**Median of CDR-limited pathways**	2025	4	-35	-60	-70
		**IMP-GS**	2020	-2	-41	-69	-45

Supplementary Table 3. The number and percentage of C1-C3 scenarios that report a given variable used and explored in this analysis. (The numbers shown are an overestimate for some variables, like Capital Cost, because some of the scenarios simply report "zero" for all years.)

	Number of scenarios			Percentage of scenarios (%)		
Variable	C1 (total = 97)	C2 (total = 134)	C3 (total=310)	C1	C2	C3
**Capacity Additions|Electricity|Coal|w/ CCS**	57	97	227	59	72	73
**Capacity Additions|Electricity|Coal|w/o CCS**	57	97	228	59	72	74
**Capacity Additions|Electricity|Gas|w/ CCS**	57	97	228	59	72	74
**Capacity Additions|Electricity|Gas|w/o CCS**	57	97	228	59	72	74
**Capacity Additions|Electricity|Non-Biomass Renewables**	57	97	228	59	72	74
**Capacity Additions|Electricity|Solar**	57	97	208	59	72	67
**Capacity Additions|Electricity|Wind**	57	97	228	59	72	74
**Capacity Additions|Electricity|Storage Capacity**	41	43	142	42	32	46
**Capital Cost|Electricity|Coal|w/ CCS**	50	57	169	52	43	55
**Capital Cost|Electricity|Coal|w/o CCS**	50	57	166	52	43	54
**Capital Cost|Electricity|Gas|w/ CCS**	50	57	169	52	43	55
**Capital Cost|Electricity|Gas|w/o CCS**	50	57	166	52	43	54
**Capital Cost|Electricity|Solar|PV**	57	92	214	59	69	69
**Capital Cost|Electricity|Solar|CSP**	45	44	143	46	33	46
**Capital Cost|Electricity|Wind|Offshore**	54	89	200	56	66	65
**Capital Cost|Electricity|Wind|Onshore**	28	74	144	29	55	46
**Capital Cost|Electricity|Storage|Battery Capacity**	0	0	0	0	0	0
**Discount rate|Economy**	0	0	0	0	0	0
**GDP|PPP**	94	119	266	97	89	86
**Emissions|CH4**	97	134	310	100	100	100
**Emissions|CH4|Energy**	75	100	223	77	75	72
**Investment|Energy Supply|Electricity|Gas**	48	94	210	49	70	68
**Investment|Energy Supply|Electricity|Gas|w/ CCS**	51	96	212	53	72	68
**Investment|Energy Supply|Electricity|Gas|w/o CCS**	51	96	212	53	72	68
**Investment|Energy Supply|Electricity|Solar**	52	96	212	54	72	68
**Investment|Energy Supply|Electricity|Wind**	52	96	212	54	72	68

**Investment|Energy Supply|Electricity|Electricity Storage**	39	76	158	40	57	51
**Consumption**	82	111	238	85	83	77
**Carbon Sequestration|CCS|Biomass**	95	124	297	98	93	96
**Carbon Sequestration|CCS|Fossil**	81	113	268	84	84	86
**Emissions|CO2|AFOLU**	97	134	307	100	100	99
**Final Energy|Non-Energy Use|Coal**	35	78	152	36	58	49
**Final Energy|Non-Energy Use|Oil**	31	77	126	32	57	41
**Final Energy|Non-Energy Use|Gas**	31	77	124	32	57	40
**Final Energy|Industry|Gases**	84	124	288	87	93	93
**Final Energy|Industry|Liquids**	84	124	288	87	93	93
**Final Energy|Industry|Solids**	84	124	288	87	93	93
**Final Energy (excl. feedstocks)|Industry|Gases**	7	7	8	7	5	3
**Final Energy (excl. feedstocks)|Industry|Liquids**	7	7	8	7	5	3
**Final Energy (excl. feedstocks)|Industry|Solids**	7	7	8	7	5	3
**Final Energy|Residential and Commercial|Gases**	76	106	269	78	79	87
**Final Energy|Residential and Commercial|Liquids**	76	106	269	78	79	87
**Final Energy|Residential and Commercial|Solids**	76	105	265	78	78	85
**Final Energy|Transportation|Gases**	77	112	278	79	84	90
**Final Energy|Transportation|Liquids**	87	118	296	90	88	95
**OM Cost|Fixed|Electricity|Coal|w/ CCS**	35	40	118	36	30	38
**OM Cost|Fixed|Electricity|Coal|w/o CCS**	37	43	130	38	32	42
**OM Cost|Fixed|Electricity|Gas|w/ CCS**	35	40	118	36	30	38
**OM Cost|Fixed|Electricity|Gas|w/o CCS**	37	43	130	38	32	42
**OM Cost|Fixed|Electricity|Solar|CSP**	34	40	116	35	30	37
**OM Cost|Fixed|Electricity|Solar|PV**	44	78	175	45	58	56
**OM Cost|Fixed|Electricity|Wind**	33	22	109	34	16	35
**OM Cost|Fixed|Electricity|Wind|Offshore**	16	57	95	16	43	31
**OM Cost|Fixed|Electricity|Wind|Onshore**	19	60	109	20	45	35
**Price|Carbon**	87	128	297	90	96	96
**Primary Energy**	94	131	310	97	98	100
**Primary Energy|Biomass**	94	131	310	97	98	100

**Primary Energy|Coal**	94	131	310	97	98	100
**Primary Energy|Gas**	94	131	310	97	98	100
**Primary Energy|Non-Biomass Renewables**	94	131	310	97	98	100
**Primary Energy|Nuclear**	94	131	310	97	98	100
**Primary Energy|Oil**	94	131	310	97	98	100
**Primary Energy|Fossil**	94	131	310	97	98	100
**Primary Energy|Fossil|w/ CCS**	76	124	278	78	93	90
**Primary Energy|Fossil|w/o CCS**	79	124	284	81	93	92
**Primary Energy|Coal|w/ CCS**	87	130	296	90	97	95
**Primary Energy|Coal|w/o CCS**	87	130	296	90	97	95
**Primary Energy|Oil|w/ CCS**	28	54	130	29	40	42
**Primary Energy|Oil|w/o CCS**	87	128	293	90	96	95
**Primary Energy|Gas|w/ CCS**	88	131	304	91	98	98
**Primary Energy|Gas|w/o CCS**	88	131	304	91	98	98
**Secondary Energy|Electricity|Coal**	94	132	310	97	99	100
**Secondary Energy|Electricity|Coal|w/ CCS**	88	132	305	91	99	98
**Secondary Energy|Electricity|Gas**	94	132	310	97	99	100
**Secondary Energy|Electricity|Gas|w/ CCS**	88	132	305	91	99	98
**Secondary Energy|Electricity|Oil**	94	132	310	97	99	100
**Secondary Energy|Electricity|Oil|w/ CCS**	22	36	127	23	27	41
**Secondary Energy|Hydrogen|Fossil**	60	87	189	62	65	61
**Secondary Energy|Hydrogen|Coal**	50	83	155	52	62	50
**Secondary Energy|Hydrogen|Oil**	7	7	18	7	5	6
**Secondary Energy|Hydrogen|Gas**	55	84	182	57	63	59

Supplementary Table 4. Gas-cluster classification and CCS-related assumptions of the model families underlying the C1 scenarios.

Model features	GEM-E3	IMAGE	GCAM	MESSAGE	AIM/CGE	POLES	WITCH	REMIND
**Percent of scenarios in gas "rebound" cluster**	100%	100%	83%	70%	0%	0%	56%	2%
**Percent of scenarios in "slow decline" cluster**	0%	0%	17%	25%	100%	100%	0%	61%
**Percent of scenarios in "fast decline" cluster**	0%	0%	0%	5%	0%	0%	44%	37%
**CCS assumption re: CO_2 storage potential**	No explicit constraint on the storage potential is applied.	Global storage potential of 5,500 $GtCO_2$ is assumed; regional constraints are placed.	Global storage potential of 7,178 $GtCO_2$ is assumed; regional constraints are placed.	No explicit constraints on the global and regional storage potentials are applied.	No explicit constraint on the storage potential is applied, but ex-ante comparison is performed with global storage potential estimates to ensure consistency.	Global and regional constraints are placed.	Default global storage potential of 11,000 $GtCO_2$ is assumed; regional constraints are indirectly placed.	Global storage potential of around 3,700 $GtCO_2$ is assumed; regional constraints are placed.
**CCS assumption re: CO_2 injection rate**	Injection rate constraints are not modelled.	Injection rate constraints are not modelled.	Injection rate constraints are not modelled.	Injection rate constraints are not modelled.	Injection rate constraints are not modelled.	Injection rate constraints are not modelled.	Injection rate constraints are not modelled.	The yearly injection rate of CO_2 is limited to 0.5% of total storage capacity due to technical and geological constraints. This corresponds to a maximum injection rate of 21.9 $GtCO_2$/yr.
**Source for CCS assumptions**	GEM-E3 model documentation[2]	Grant et al. 2022, Table 1[3]	Grant et al. 2022, Table 1[3]	Grant et al. 2022, Table 1[3]	Grant et al. 2022, Table 1[3]	Koelb et al. 2014[4]	Grant et al. 2022, Table 1[3]	Grant et al. 2022, Table 1[3]

SUPPLEMENTARY FIGURES

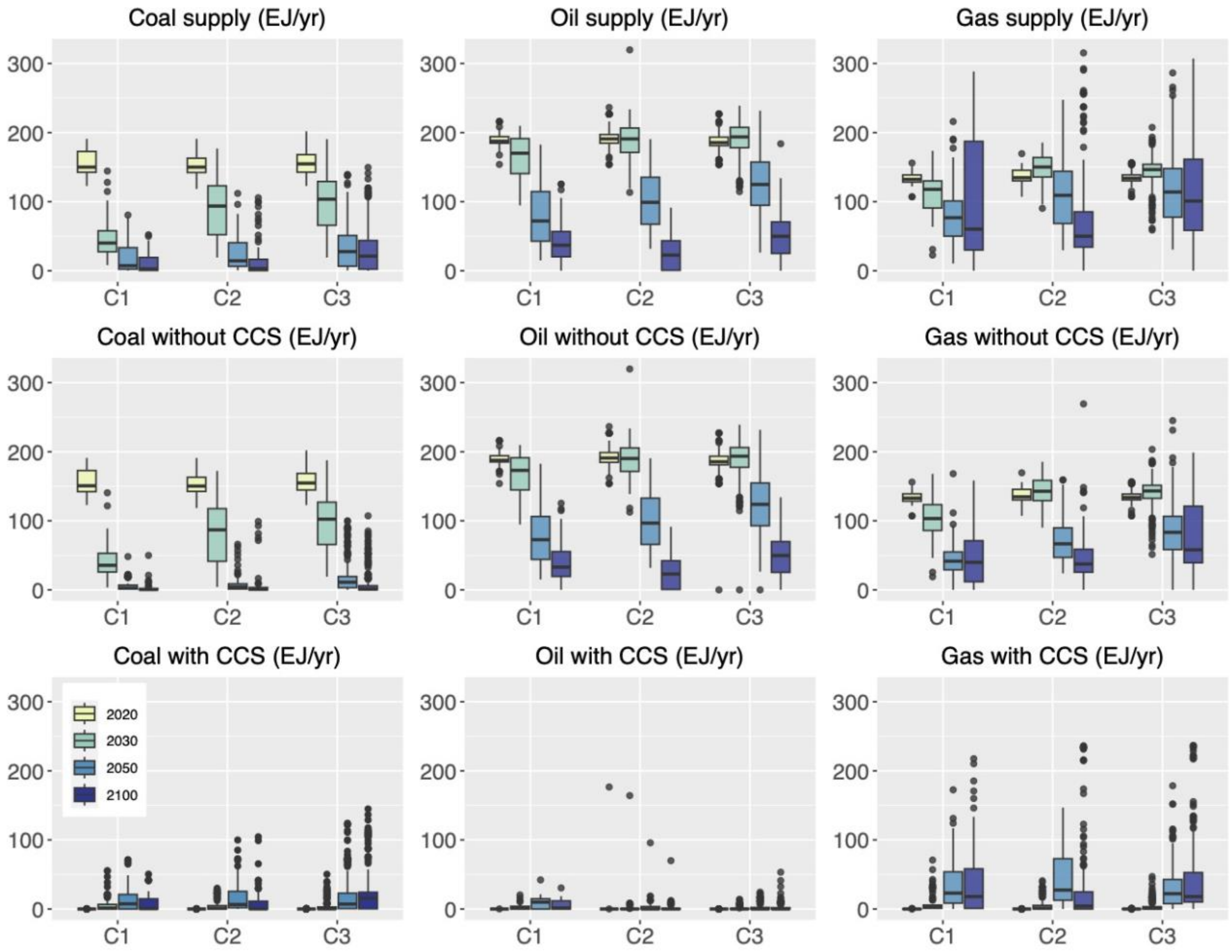

Supplementary Fig. 1. Boxplot distributions of the annual primary energy supply (exajoules per year) from coal, oil, and gas in 2020, 2030, 2050, and 2100 modelled in the AR6 C1-C3 scenarios. The top row shows total primary energy supply from each fuel, the middle row shows the supply that is not coupled to CCS at the point of combustion, and the bottom row shows the supply coupled to CCS at the point of combustion. The horizontal center line depicts the median, the box spans the interquartile range (IQR) between the 25th percentile (Q1) and 75th percentile (Q3), the lower whisker represents the minimum value or Q1 - 1.5 x IQR (whichever is larger), and the upper whisker represents the maximum value or Q3 + 1.5 x IQR (whichever is smaller). Outliers are depicted as black dots.

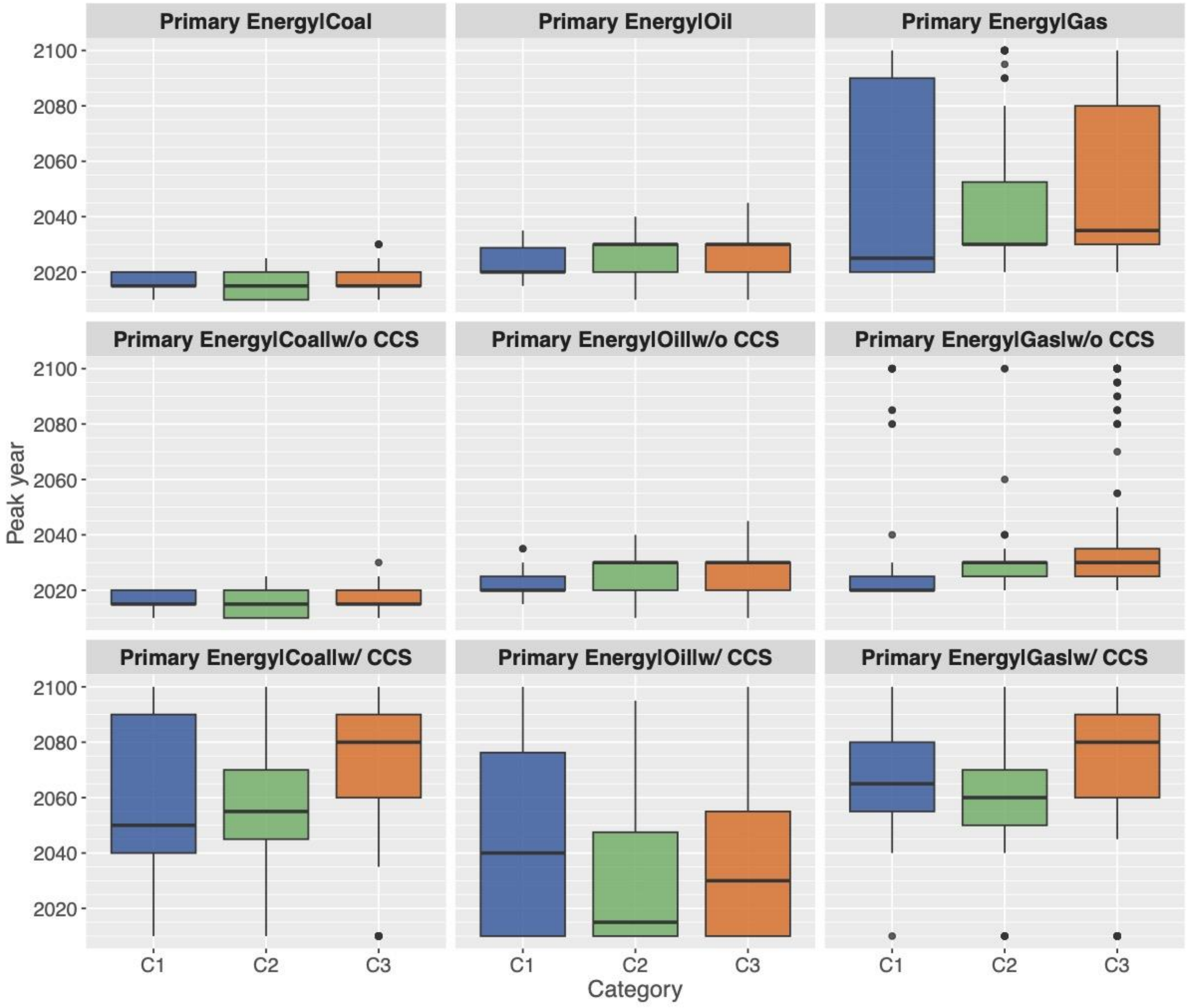

Supplementary Fig. 2. Boxplot distributions of the year (data available at 5-year intervals) in which global primary energy supply by coal, oil, or gas peaks between 2010-2100 in the AR6 C1-C3 scenarios. The top row shows total supply for each fuel, the middle row shows the supply that is not coupled to CCS at the point of combustion, and the bottom row shows the supply coupled to CCS at the point of combustion. The horizontal center line depicts the median, the box spans the interquartile range (IQR) between the 25th percentile (Q1) and 75th percentile (Q3), the lower whisker represents the minimum value or Q1 - 1.5 x IQR (whichever is larger), and the upper whisker represents the maximum value or Q3 + 1.5 x IQR (whichever is smaller). Outliers are depicted as black dots.

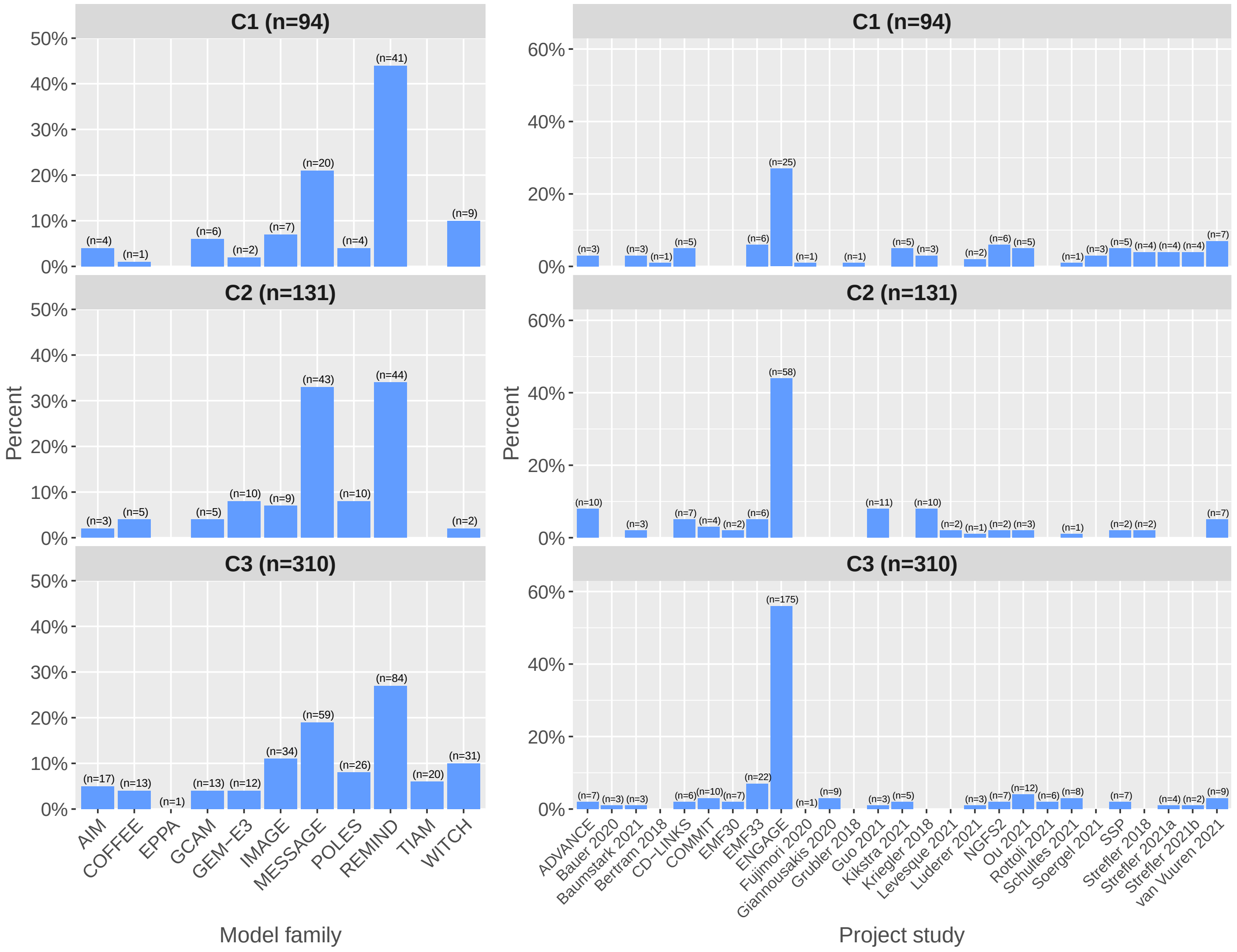

Supplementary Fig. 3. The percentage (and number) of individual scenarios from each model family (left column) and project study (right column) that report the variables “Primary Energy|Coal”, “Primary Energy|Oil”, and “Primary Energy|Gas” within each category for C1-C3 vetted scenarios in the IPCC AR6 database.

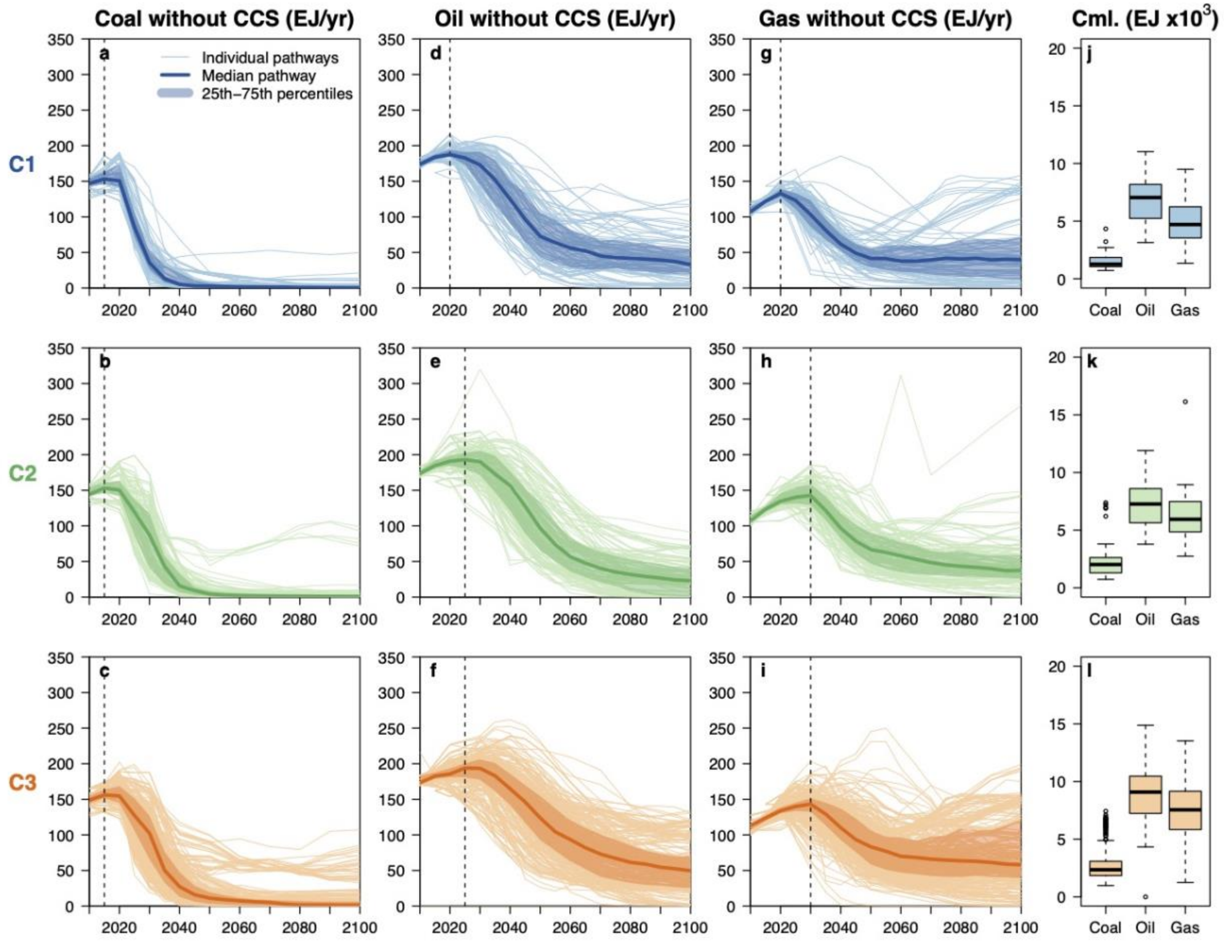

Supplementary Fig. 4. Global primary energy supply (in exajoules, EJ) from coal, oil, and fossil gas that is not coupled to carbon capture and storage (CCS) at the point of combustion, as modelled by the IPCC AR6 mitigation scenarios consistent with the Paris Agreement. In subplots a-i, the 2010-2100 annual timeseries of the following are plotted at each 5-year interval within a given temperature category (C1-C3): individual pathways (light lines); median values (dark lines); and the 25th-75th percentiles (shaded ranges). Subplots j-l show the boxplot distributions of the 2020-2100 cumulative supply across the scenario ensemble within each temperature category. (Not all model-scenarios report the CCS-breakdown portions, especially for oil with CCS.) The horizontal center line depicts the median, the box spans the interquartile range (IQR) between the 25th percentile (Q1) and 75th percentile (Q3), the lower whisker represents the minimum value or Q1 - 1.5 x IQR (whichever is larger), and the upper whisker represents the maximum value or Q3 + 1.5 x IQR (whichever is smaller).

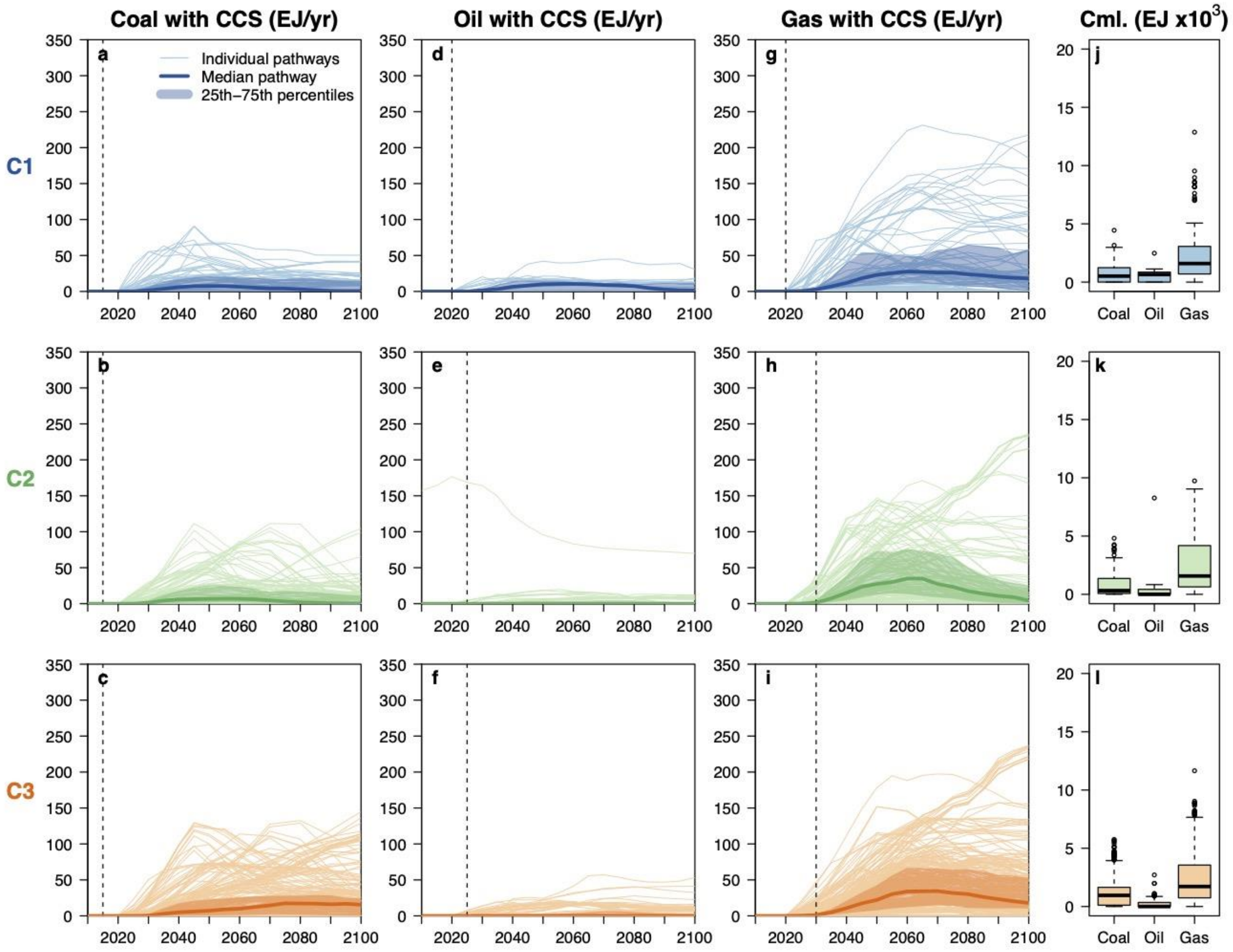

Supplementary Fig. 5. As in Supplementary Fig. 4 but for coal, oil, and fossil gas supply that is coupled to CCS at the point of combustion.

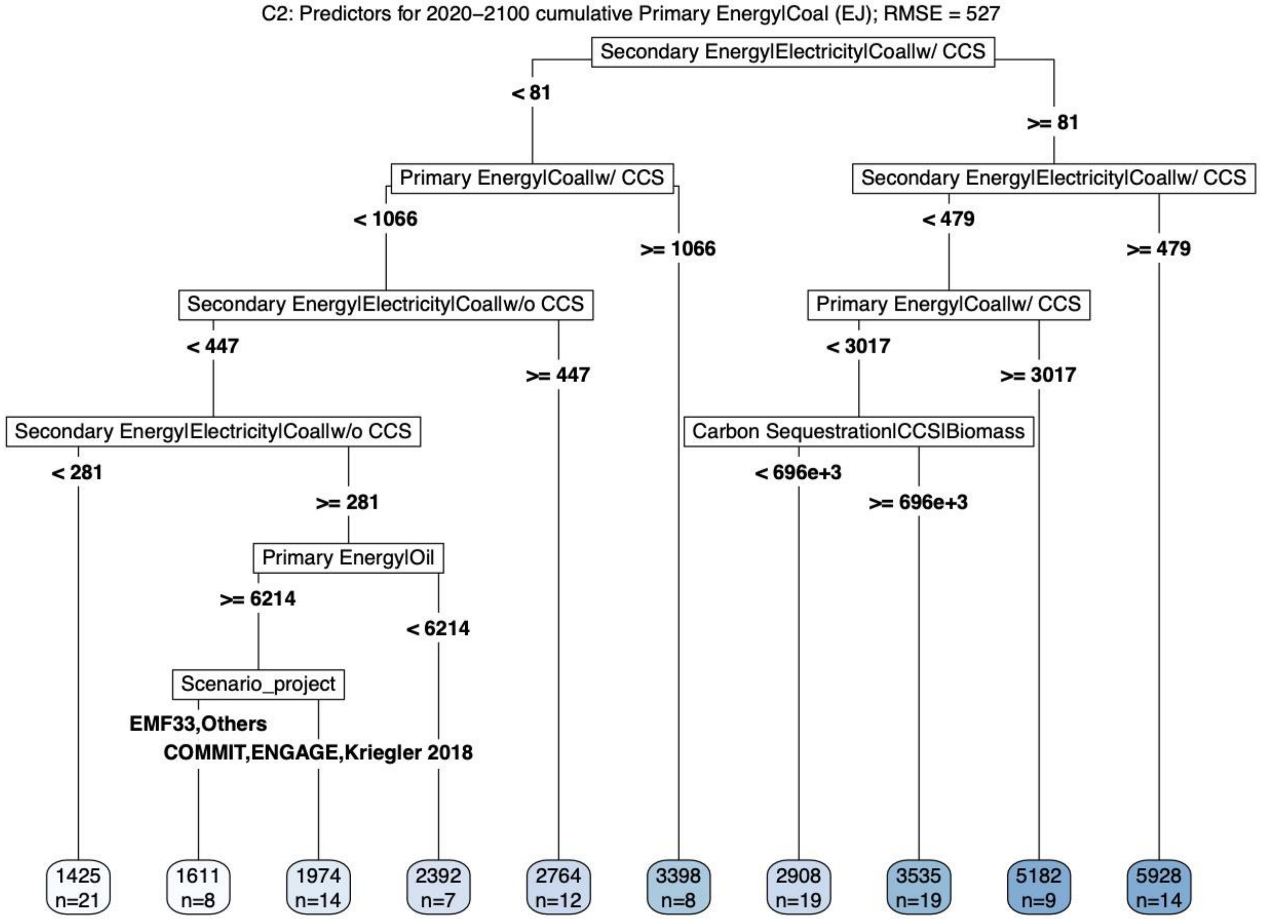

Supplementary Fig. 6. CART results for predicting the 2020-2100 cumulative primary energy supply by coal modelled in the C2 scenarios. The blue boxes show the average value of cumulative coal supply (in units of exajoules, EJ) across the scenarios within each given "leaf node" *(note that they do not necessarily appear in increasing order from left to right)*. For example, scenarios with cumulative coal supply around 5,928 EJ are associated with electricity generation from coal coupled to CCS greater than or equal to 479 EJ. The units of the 2020-2100 cumulative values of the independent variables shown are exajoules for primary and secondary energy, and million tonnes of CO_2 for emissions and carbon sequestration. Note that "Solids", "Liquids", or "Gases" reported under Final Energy can also include non-fossil fuel components, and that negative AFOLU emissions represent CO_2 removal.

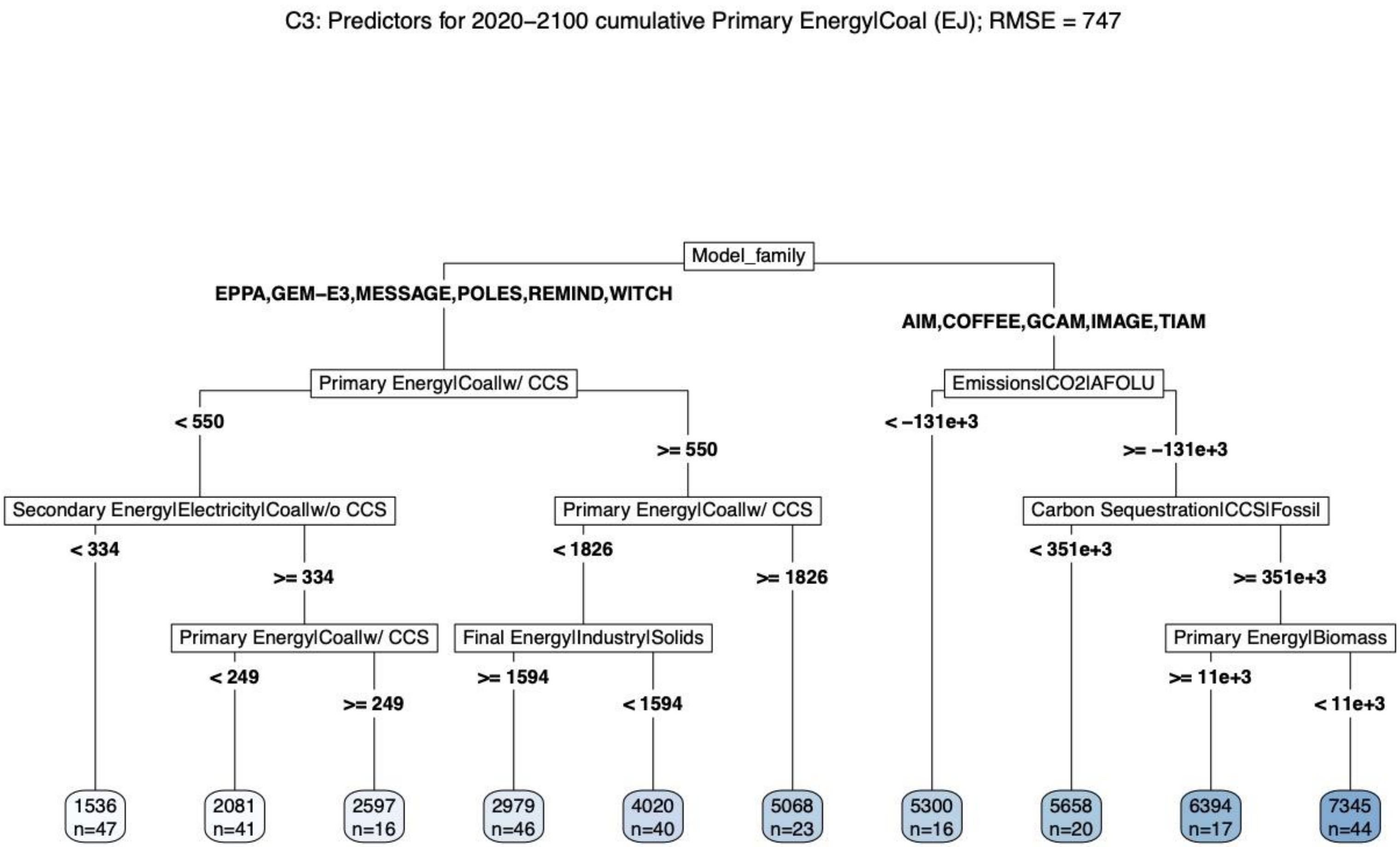

Supplementary Fig. 7. As described in Supplementary Fig. 6 but for C3-coal.

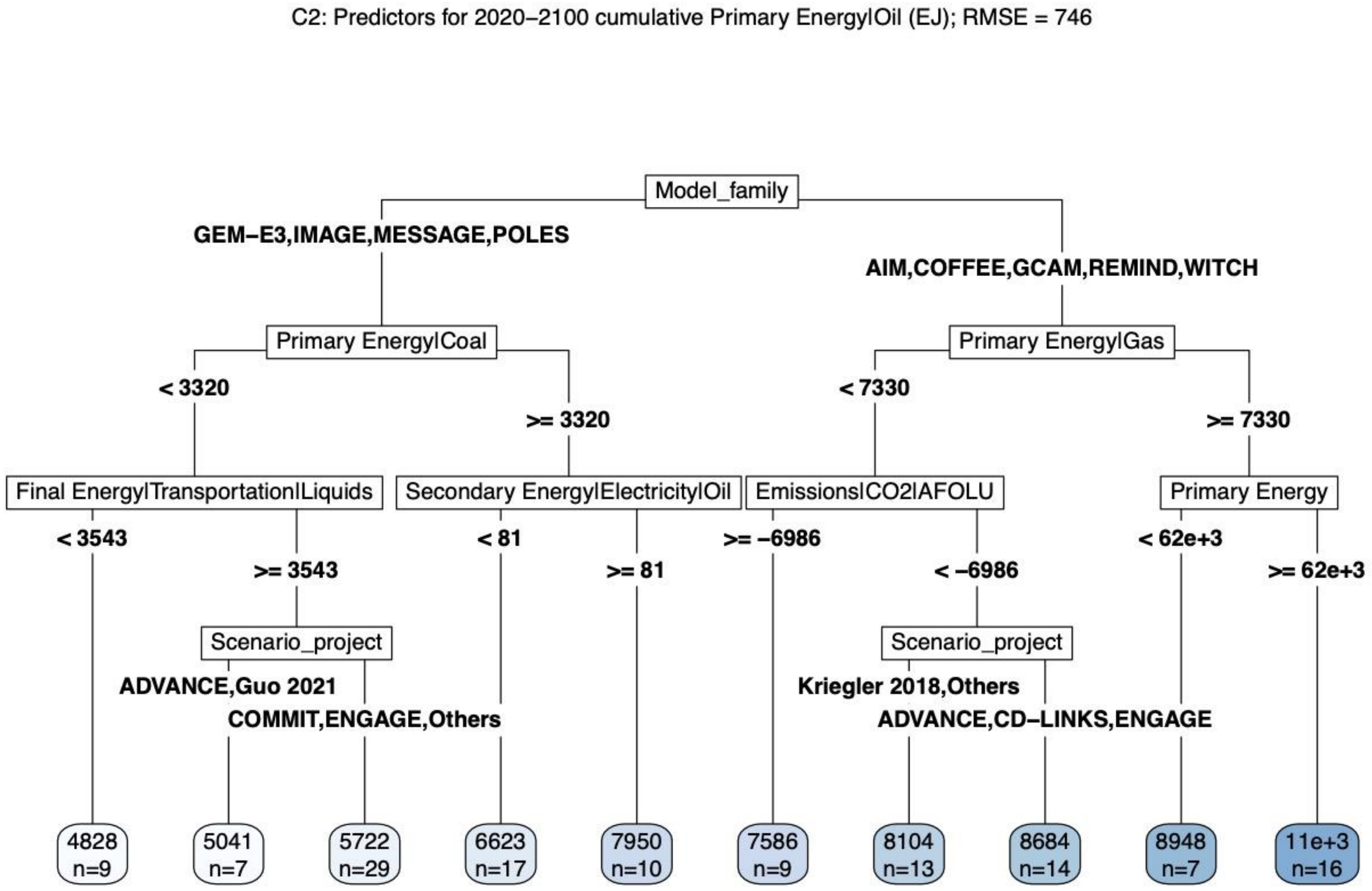

Supplementary Fig. 8. As described in Supplementary Fig. 6 but for C2-oil.

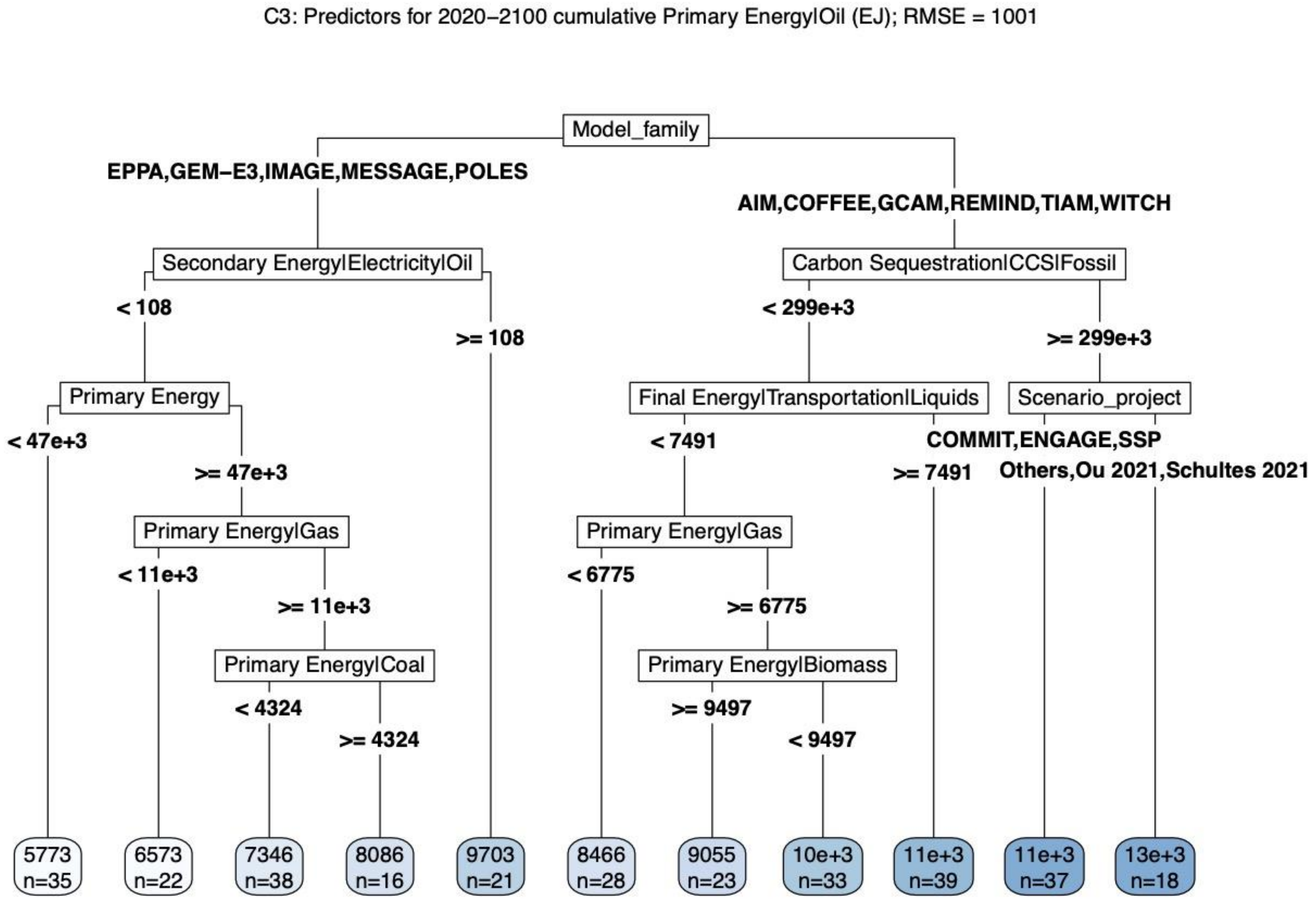

Supplementary Fig. 9. As described in Supplementary Fig. 6 but for C3-oil.

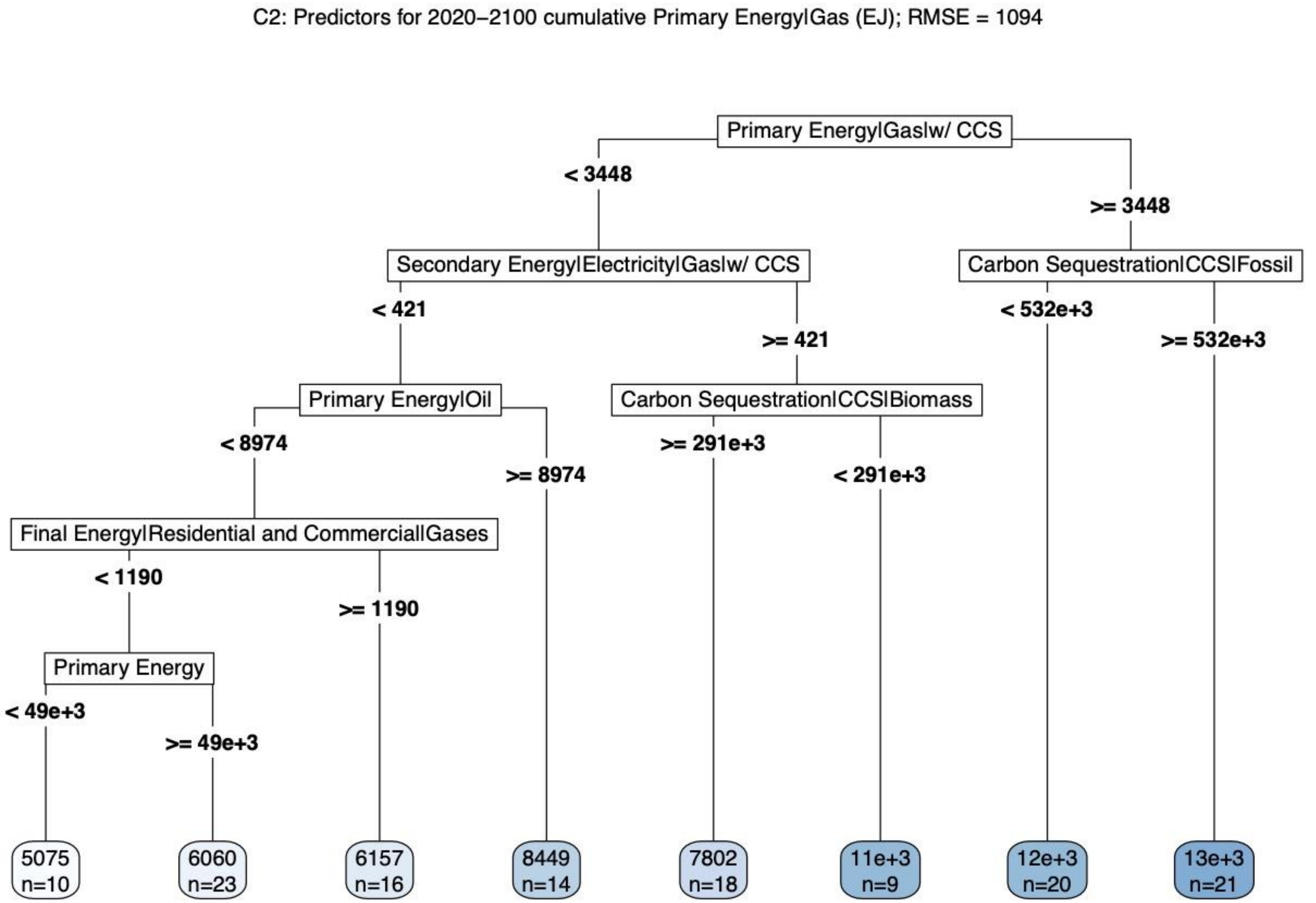

Supplementary Fig. 10. As described in Supplementary Fig. 6 but for C2-gas.

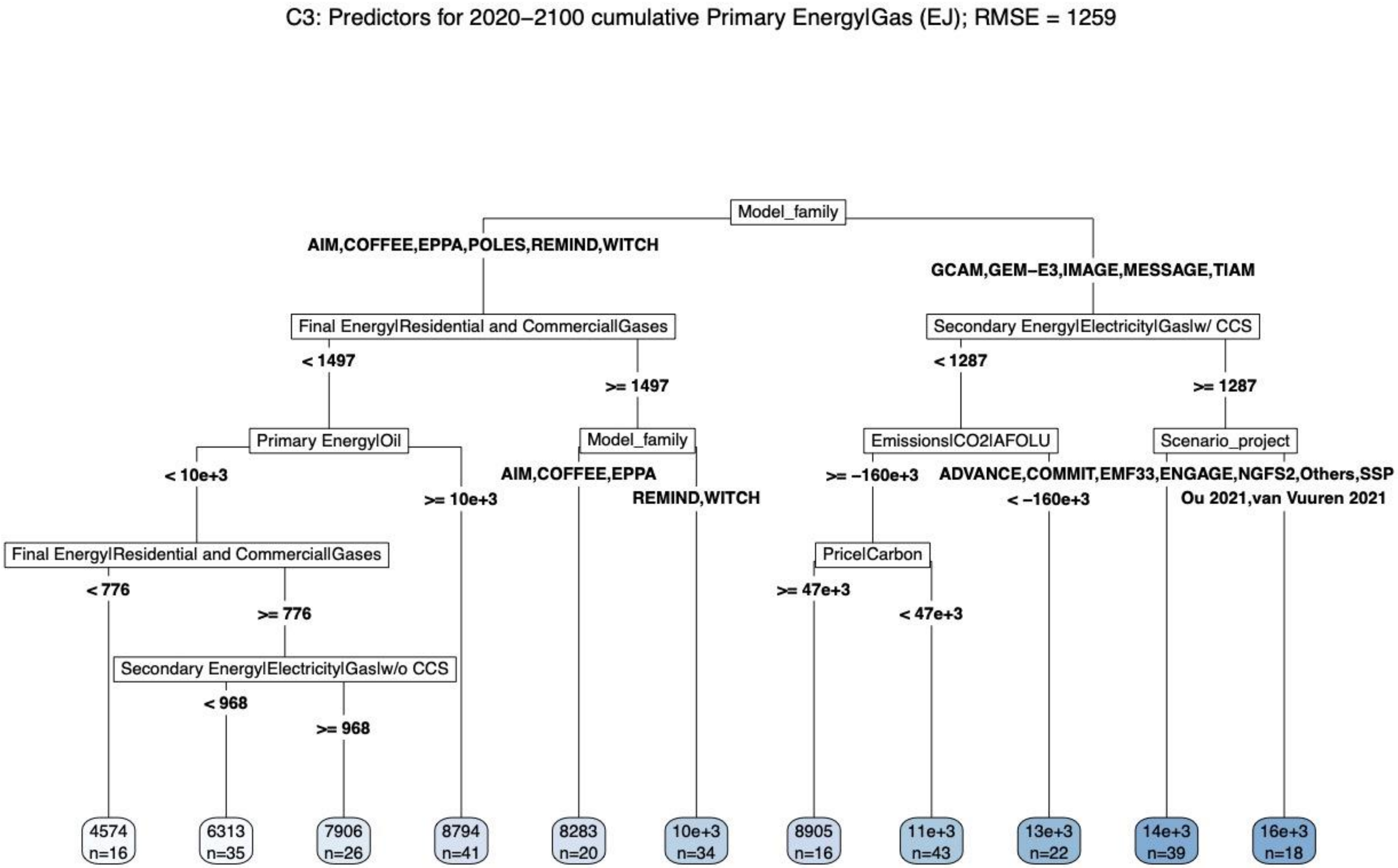

Supplementary Fig. 11. As described in Supplementary Fig. 6 but for C3-gas.

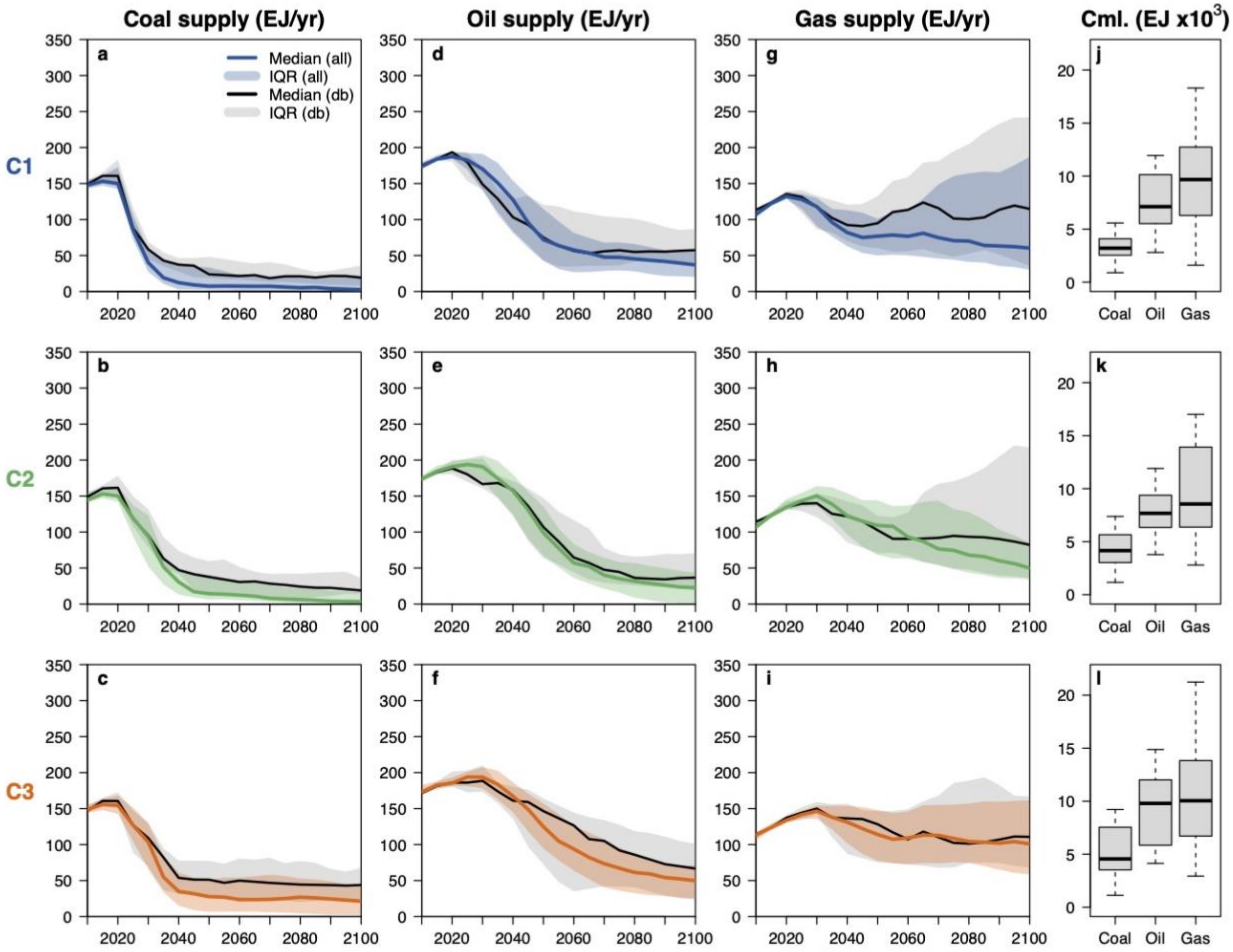

Supplementary Fig. 12. As in Figure 1, but also showing the median pathway (black line) and interquartile ranges (shaded grey area) if only the scenarios with minimum and maximum cumulative 2020-2100 supply values for a given model family, fuel, and category are included in the calculations. Note that this is one simple approach to debias the scenario ensemble with respect to over- and under-representation by certain model families; there is limited practice in and consensus on bias correction of climate mitigation scenario ensembles[9]. The horizontal center line depicts the median, the box spans the interquartile range (IQR) between the 25th percentile (Q1) and 75th percentile (Q3), the lower whisker represents the minimum value or Q1 - 1.5 x IQR (whichever is larger), and the upper whisker represents the maximum value or Q3 + 1.5 x IQR (whichever is smaller).

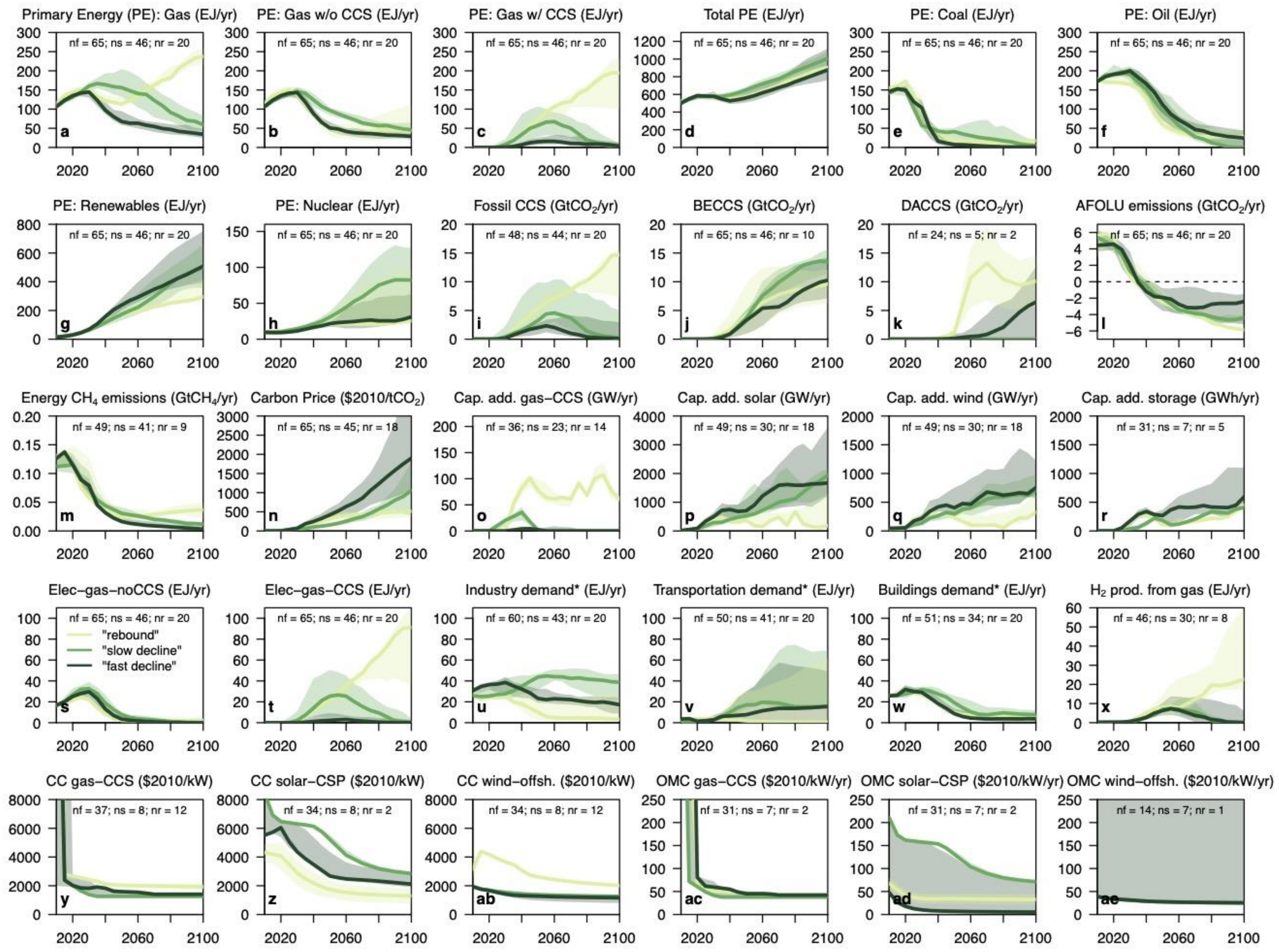

Supplementary Fig. 13. As in Figure 4 but for the C2 scenarios.

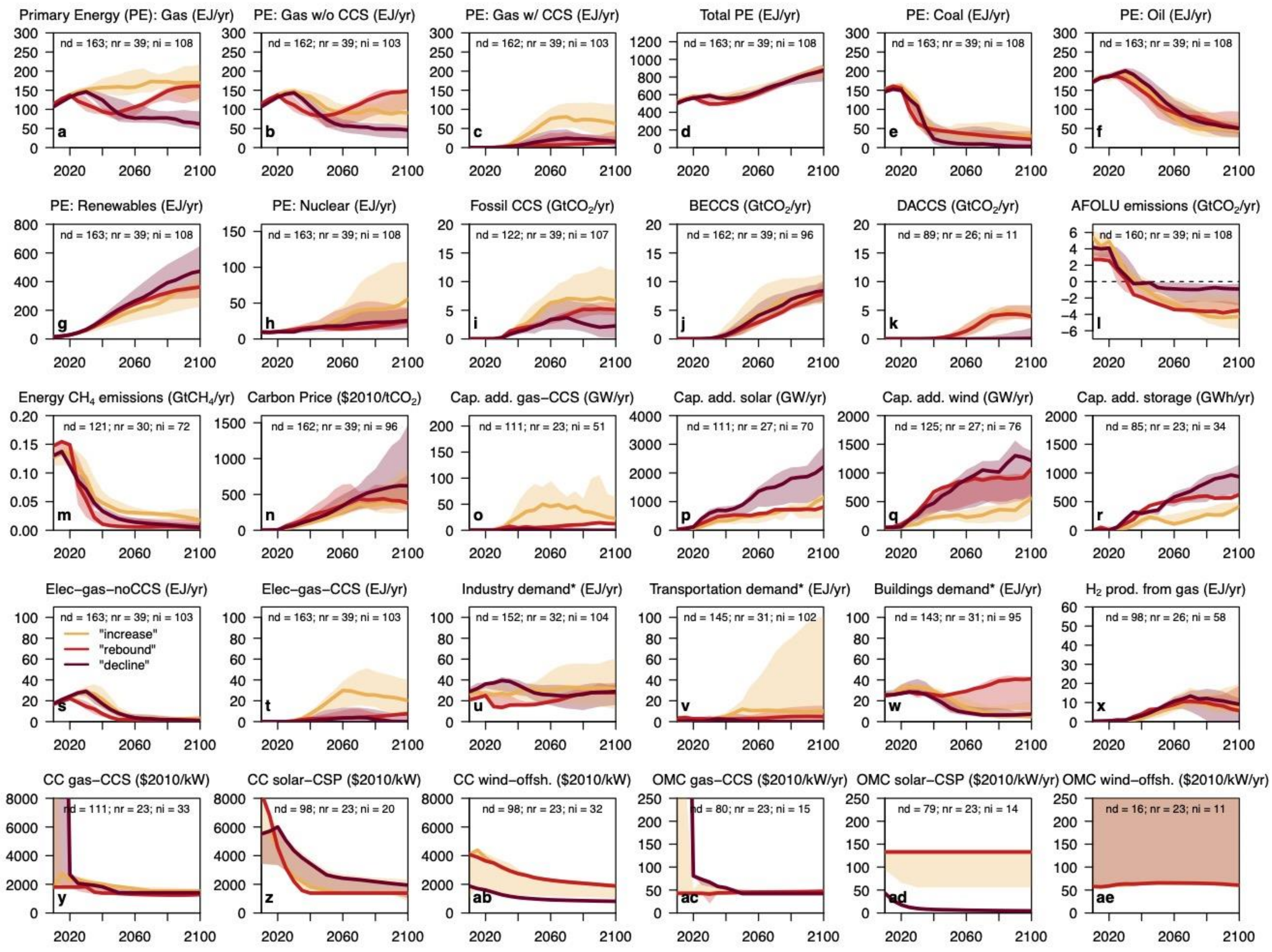

Supplementary Fig. 14. As in Figure 4 but for the C3 scenarios.

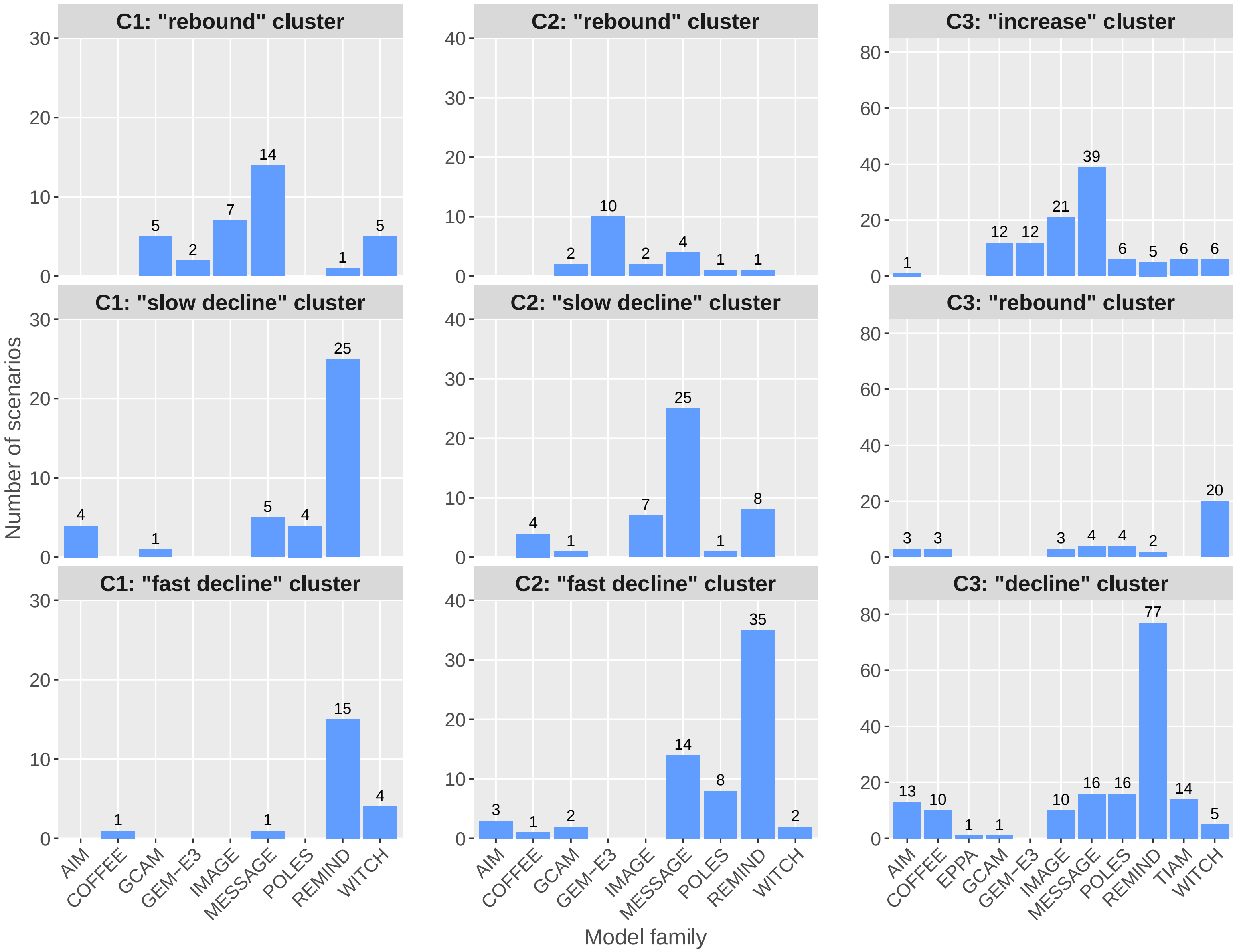

Supplementary Fig. 15. Number of scenarios from different model families in each of the gas clusters in Figures 4 and S13-S14.

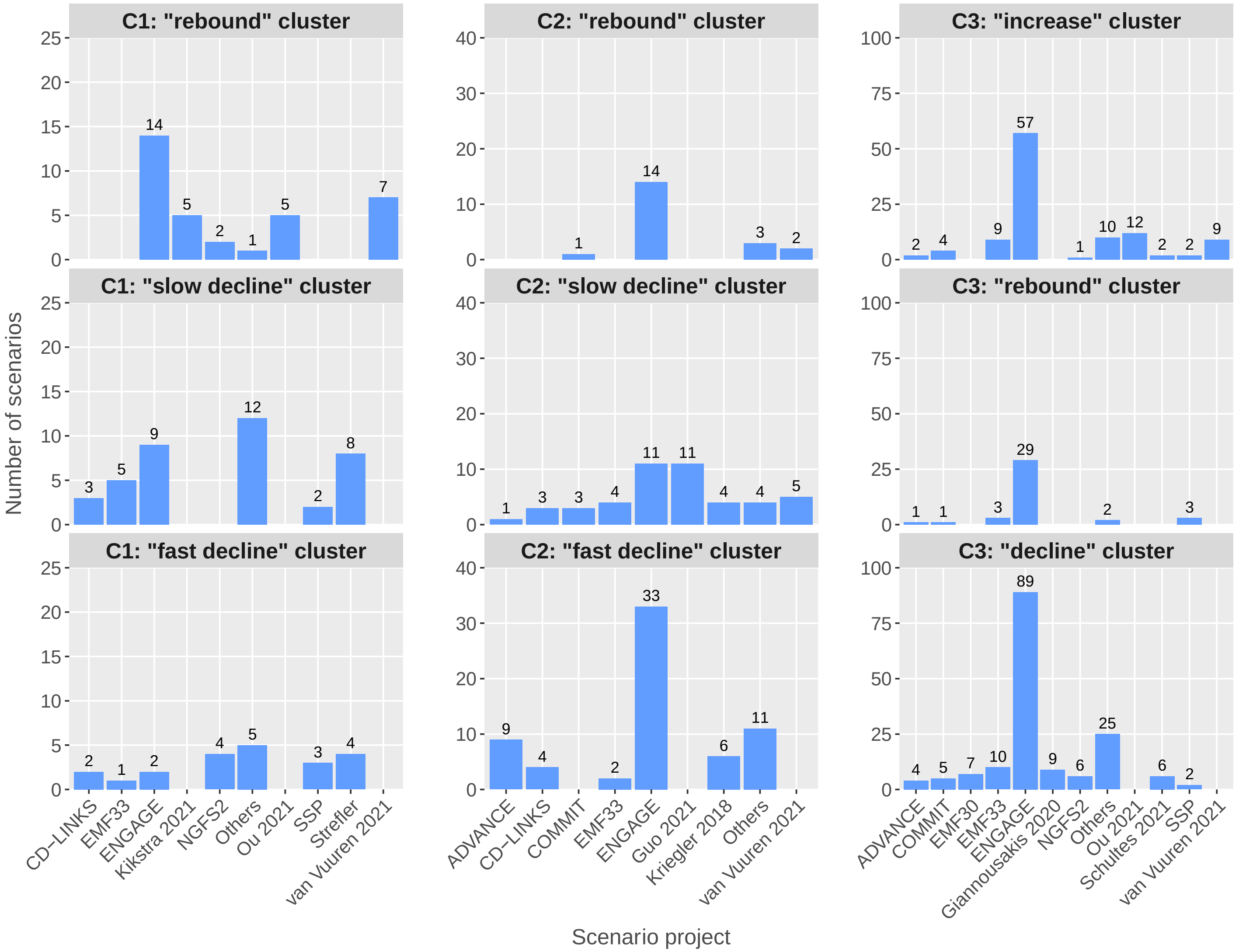

Supplementary Fig. 16. Number of scenarios from different major scenario projects in each of the gas clusters in Figure 4 and Supplementary Figures 13-14. To allow easy visualization, the projects with a relatively small number of scenarios (Supplementary Fig. 3) have been grouped into the “Others” category here.

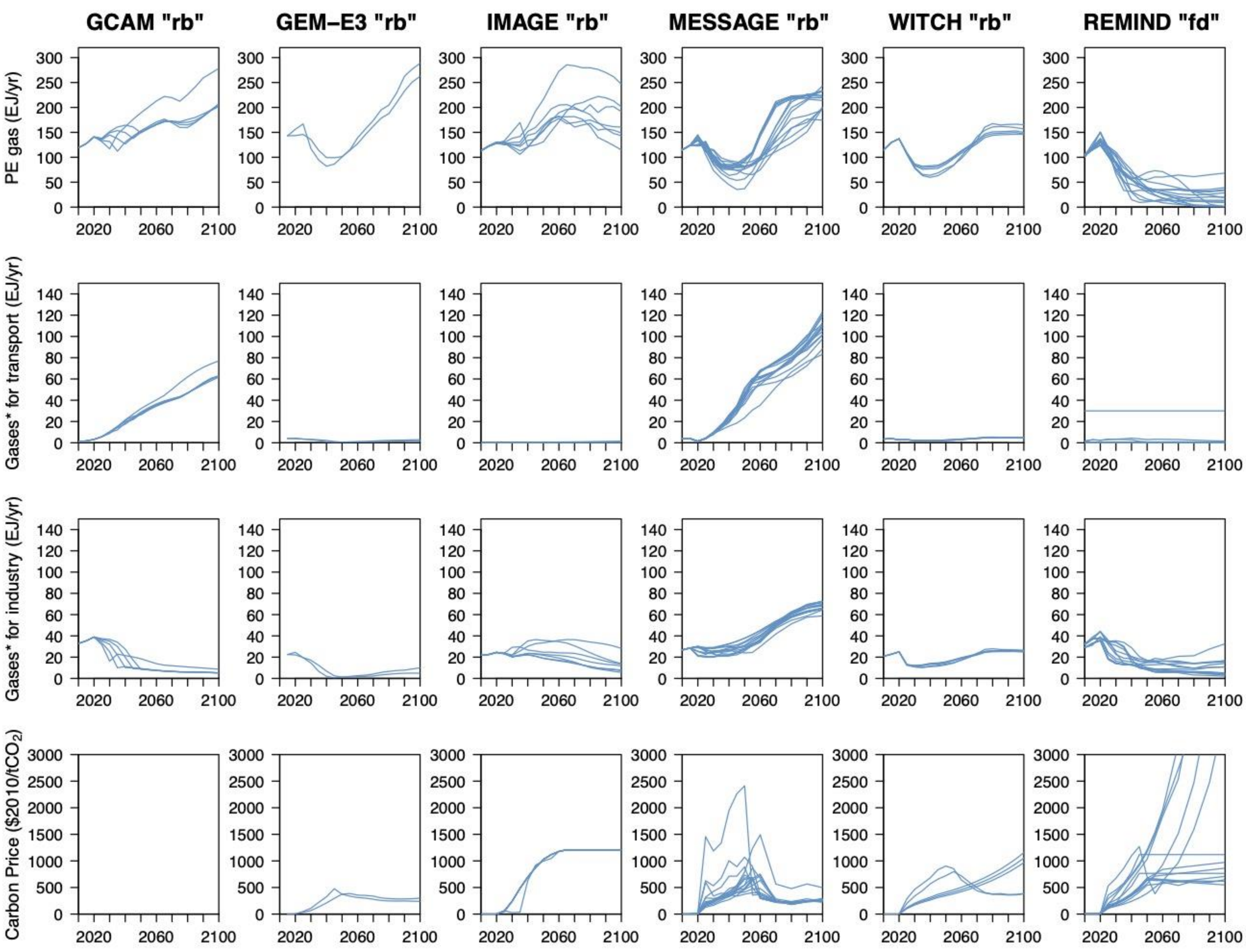

Supplementary Fig. 17. Individual pathways of select variables for the C1 scenarios grouped into the "rebound (rb)" or "fast decline (fd)" gas clusters for select model families (see Supplementary Fig. 15). (We note that the GCAM scenarios from the Ou et al. 2021 study were from a sensitivity study, but since the modelled outputs for many variables, including gas supply, show similar trends to other C1 "rebound" scenarios, we include them in our analysis here.)

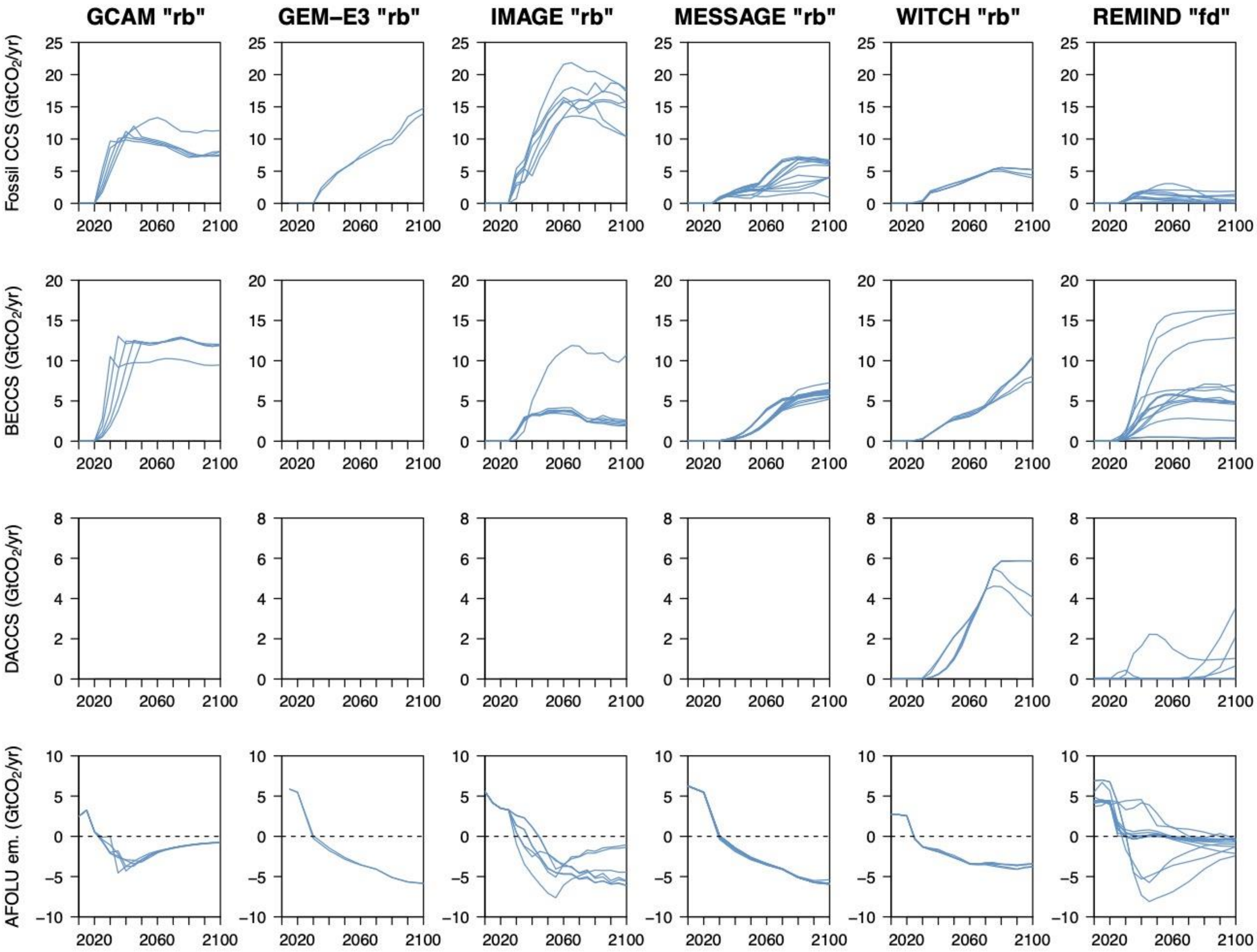

Supplementary Fig. 18. Individual pathways of select variables for the C1 scenarios grouped into the "rebound (rb)" or "fast decline (fd)" gas clusters for select model families (see Supplementary Fig. 15).

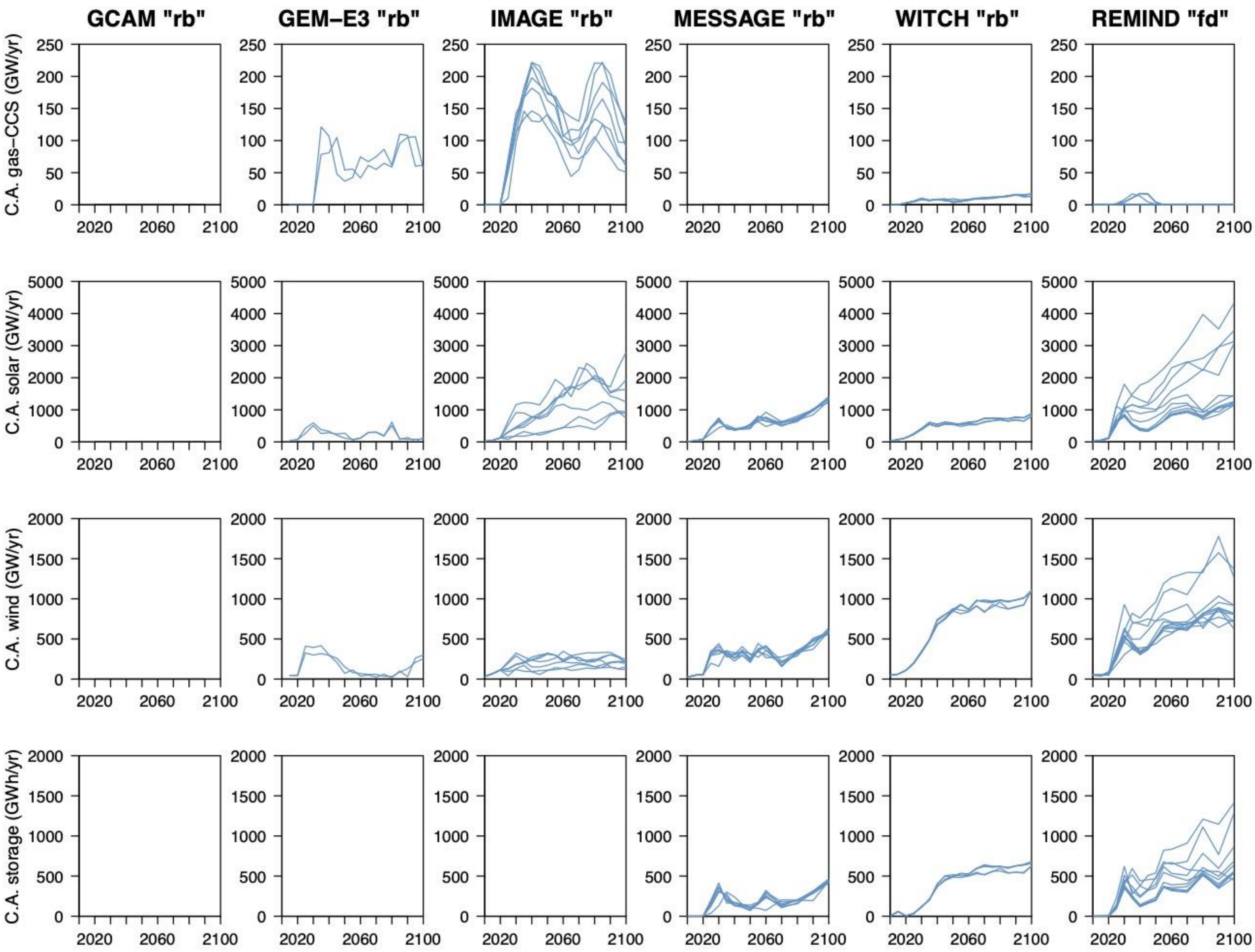

Supplementary Fig. 19. Individual pathways of select variables for the C1 scenarios grouped into the "rebound (rb)" or "fast decline (fd)" gas clusters for select model families (see Supplementary Fig. 15).

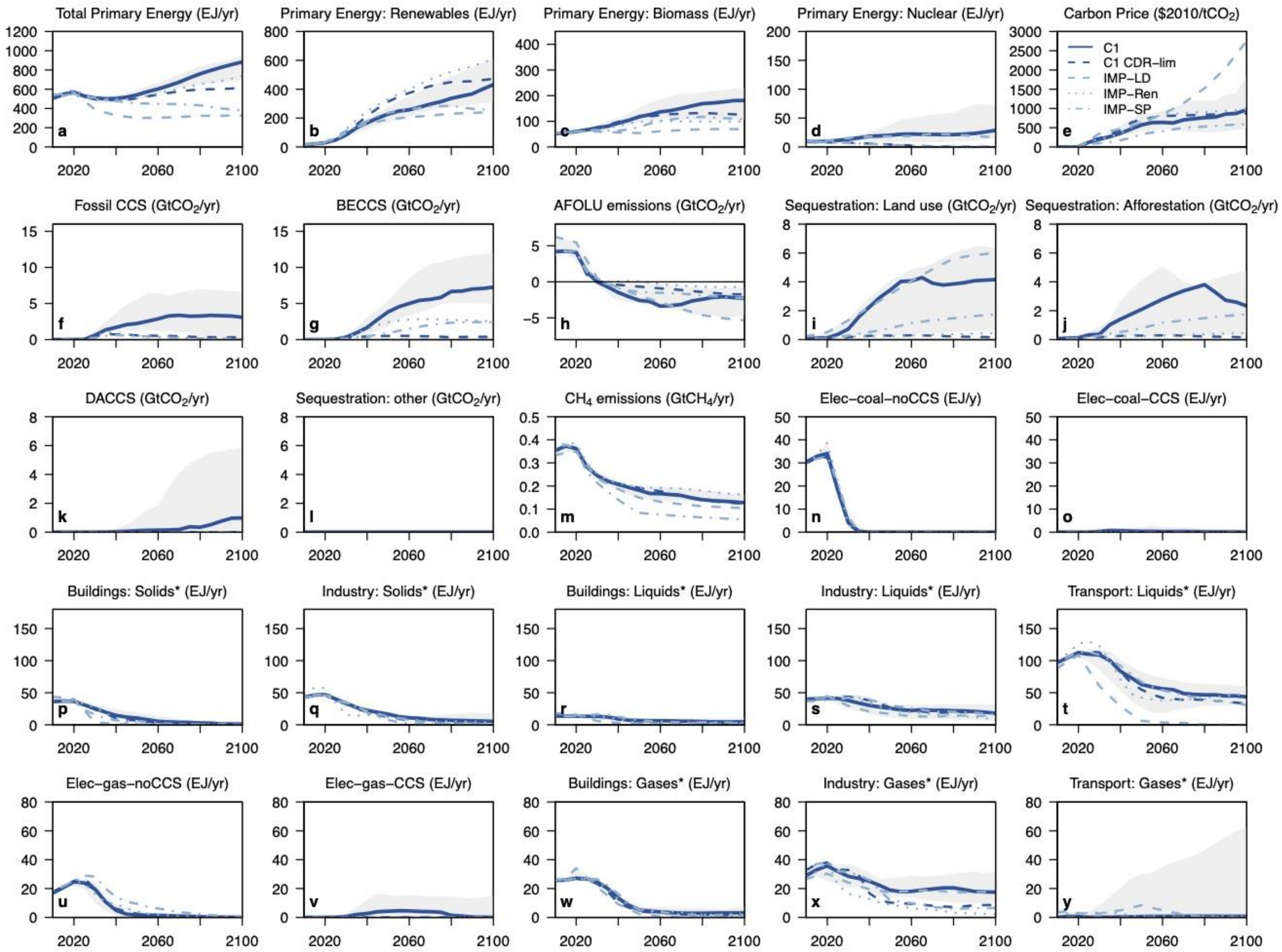

Supplementary Fig. 20. 2015-2100 values for select variables for the C1 pathways shown in Figure 5: (1) the median and interquartile ranges of all scenarios; (2) the median of scenarios constrained by limits on BECCS, AR, and DACCS based on expert consensus of their future CDR potential; and (3) individual illustrative mitigation pathways (IMP-LD, -SP, -Ren).

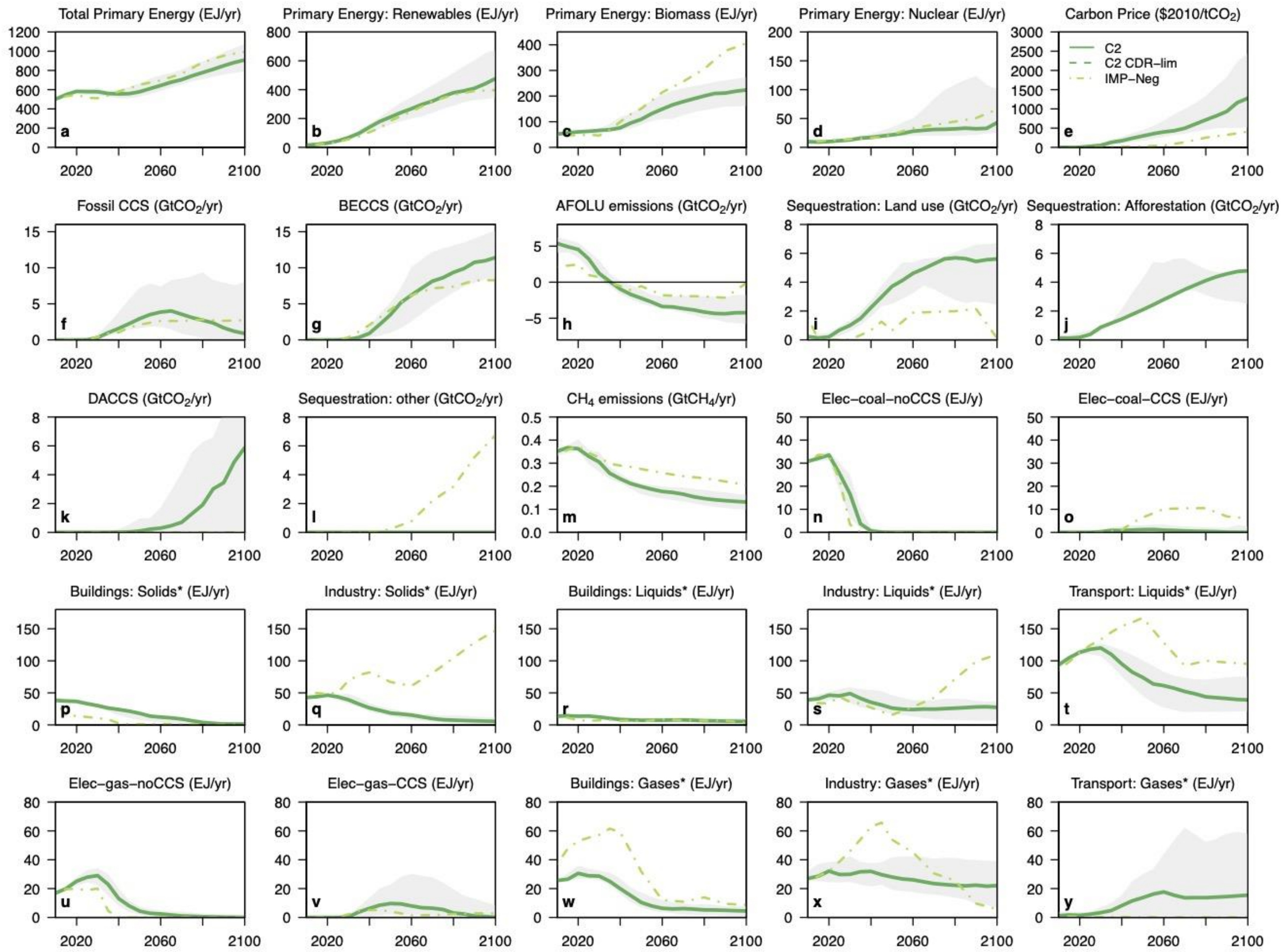

Supplementary Fig. 21. 2015-2100 values for select variables for the C2 pathways shown in Figure 5: (1) the median and interquartile ranges of all scenarios; and (2) the illustrative C2 mitigation pathway (IMP-GS). All C2 scenarios rely on BECCS, AR, and/or DACCS at levels that exceed the expert consensus of their future, respective CDR potential.

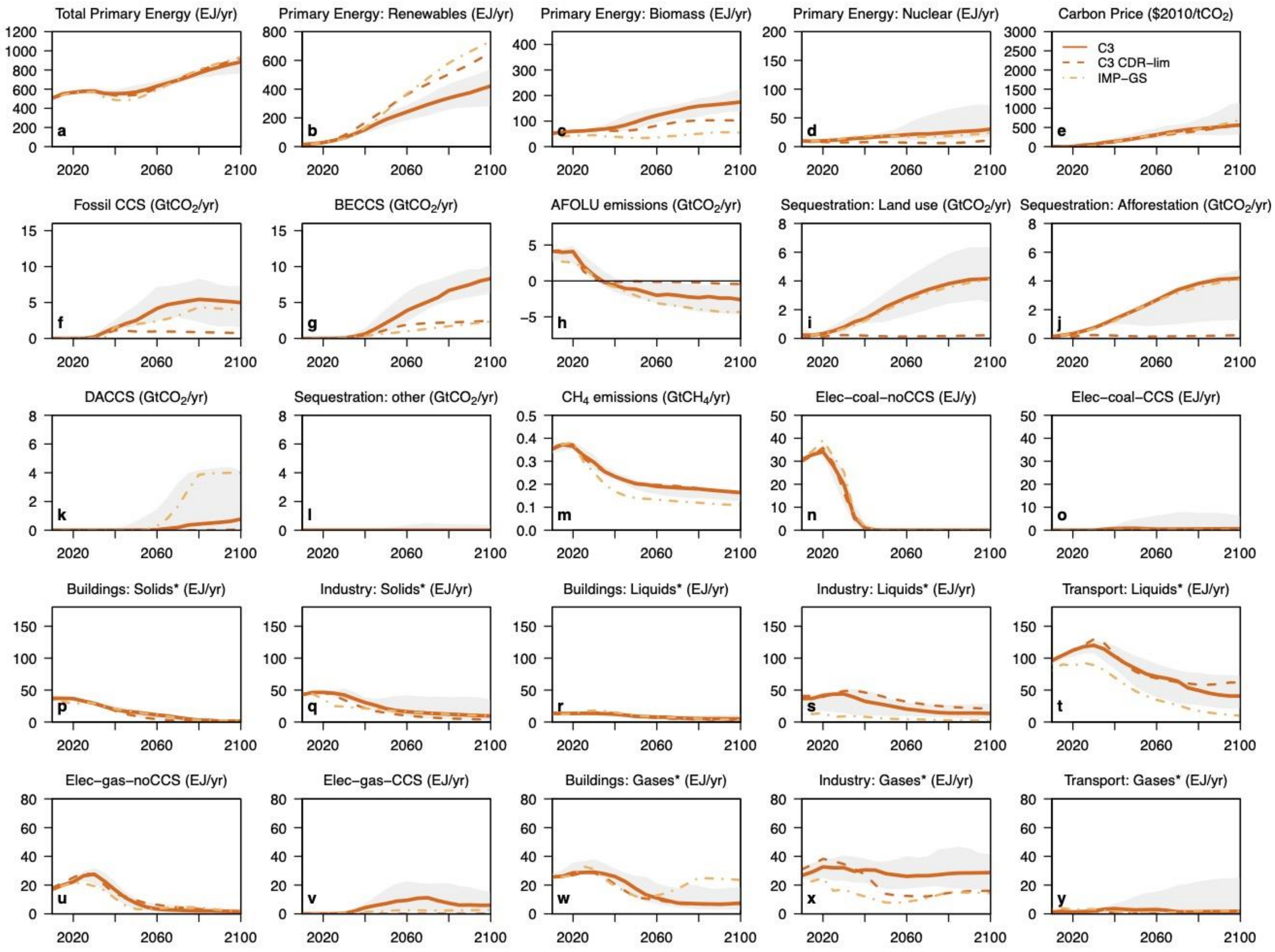

Supplementary Fig. 22. 2015-2100 values for select variables for the C3 pathways shown in Figure 5: (1) the median and interquartile ranges of all scenarios; (2) the median of scenarios constrained by limits on BECCS, AR, and DACCS based on expert consensus of their future CDR potential; and (3) the C3 illustrative mitigation pathway (IMP-GS).

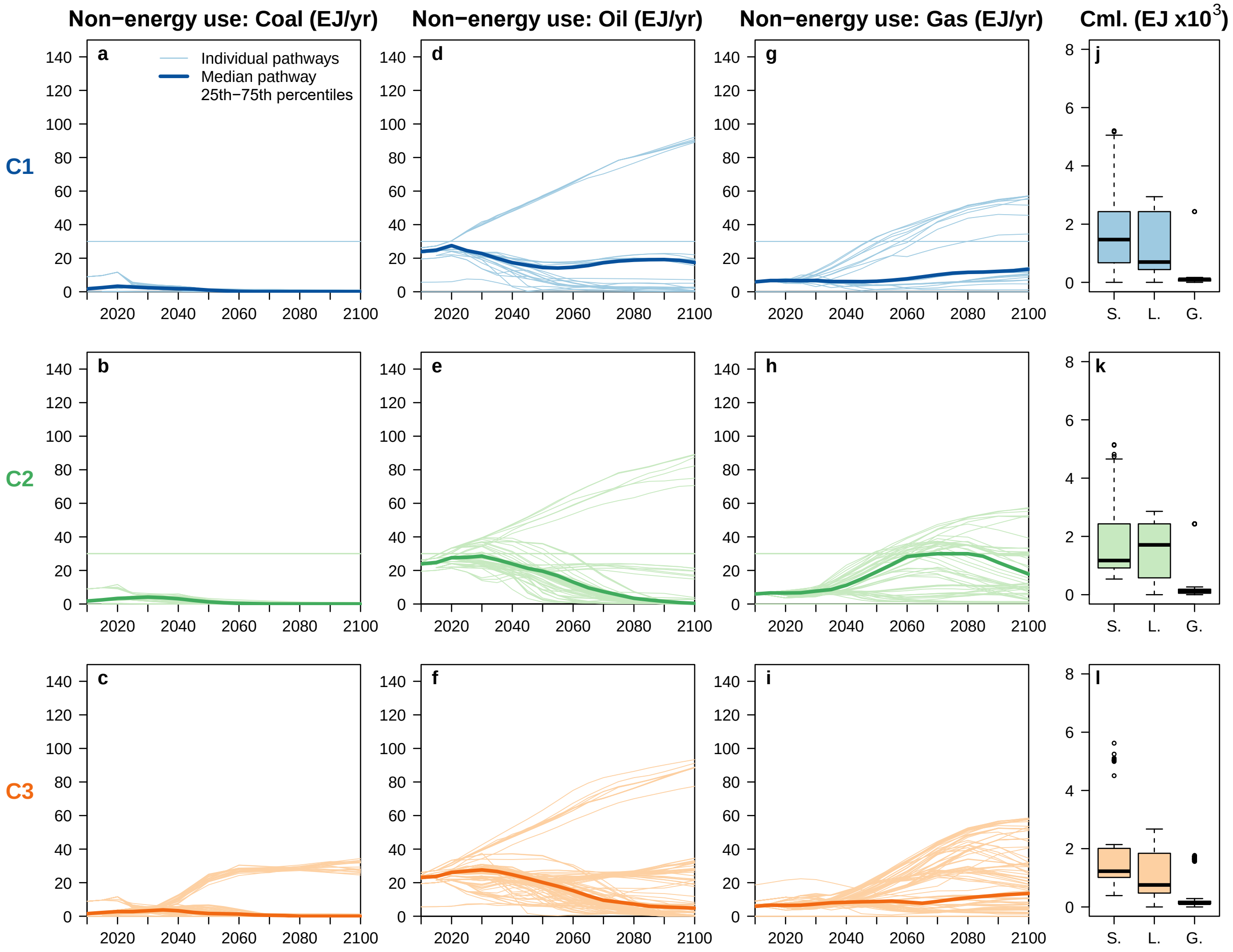

Supplementary Fig. 23. Non-energy use of coal, oil, and gas ("Final Energy|Non-Energy Use|xx", in exajoules, EJ) as modelled by the IPCC AR6 mitigation scenarios consistent with limiting warming to 1.5°C or 2°C. In subplots a-i, the 2010-2100 annual timeseries of the following are plotted at each 5-year interval within a given temperature category (C1-C3): individual pathways (light lines); median values (dark lines); and the 25th-75th percentiles (shaded ranges). Subplots j-l show the boxplot distributions of the 2020-2100 cumulative values across the scenario ensemble within each temperature category. The horizontal center line depicts the median, the box spans the interquartile range (IQR) between the 25th percentile (Q1) and 75th percentile (Q3), the lower whisker represents the minimum value or Q1 - 1.5 x IQR (whichever is larger), and the upper whisker represents the maximum value or Q3 + 1.5 x IQR (whichever is smaller).

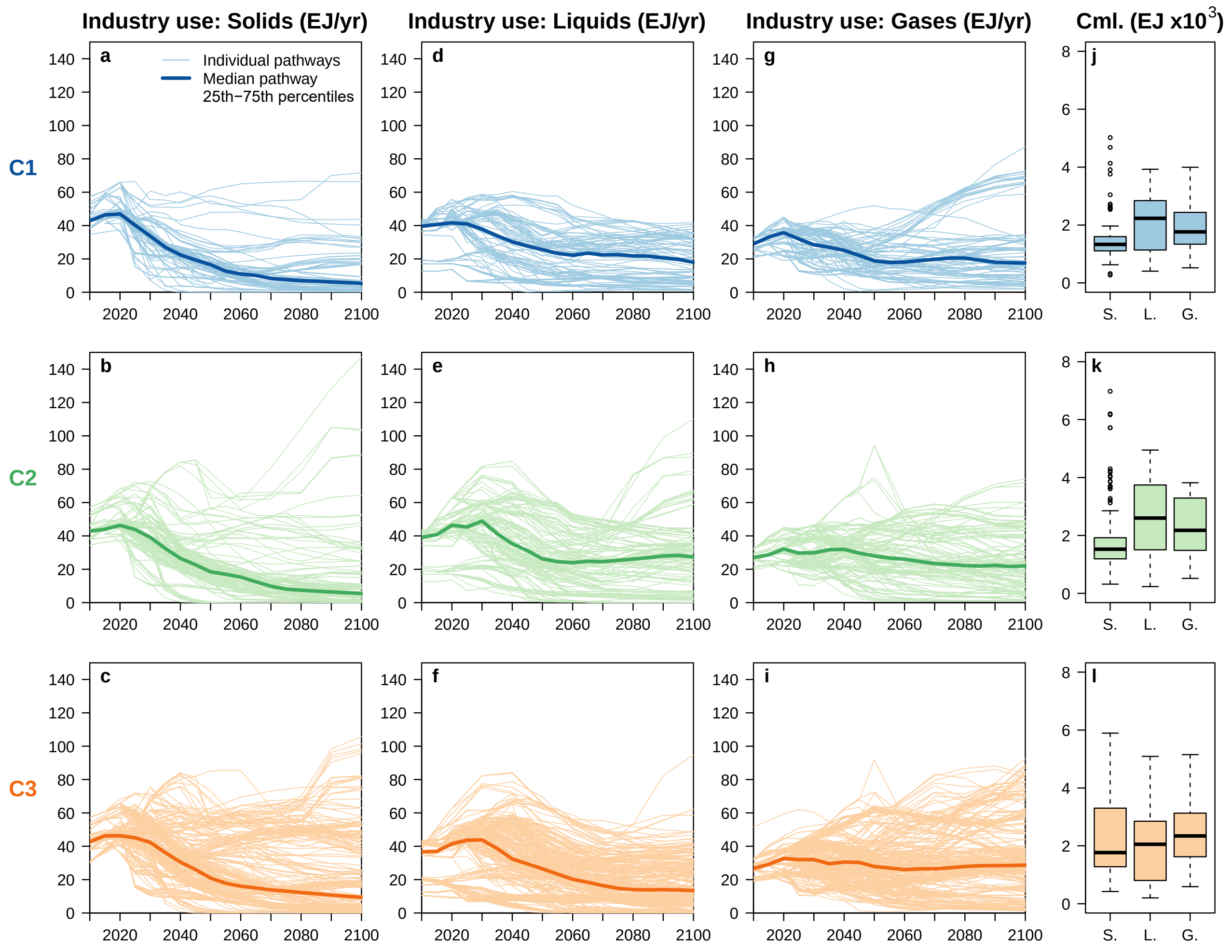

Supplementary Fig. 24. Non-energy industry use of fossil fuel- and bio-derived solids, liquids, and gases ("Final Energy|Industry|xx", in exajoules, EJ) as modelled by the IPCC AR6 mitigation scenarios consistent with limiting warming to 1.5°C or 2°C. In subplots a-i, the 2010-2100 annual timeseries of the following are plotted at each 5-year interval within a given temperature category (C1-C3): individual pathways (light lines); median values (dark lines); and the 25th-75th percentiles (shaded ranges). Subplots j-l show the boxplot distributions of the 2020-2100 cumulative values across the scenario ensemble within each temperature category. The horizontal center line depicts the median, the box spans the interquartile range (IQR) between the 25th percentile (Q1) and 75th percentile (Q3), the lower whisker represents the minimum value or Q1 - 1.5 x IQR (whichever is larger), and the upper whisker represents the maximum value or Q3 + 1.5 x IQR (whichever is smaller).

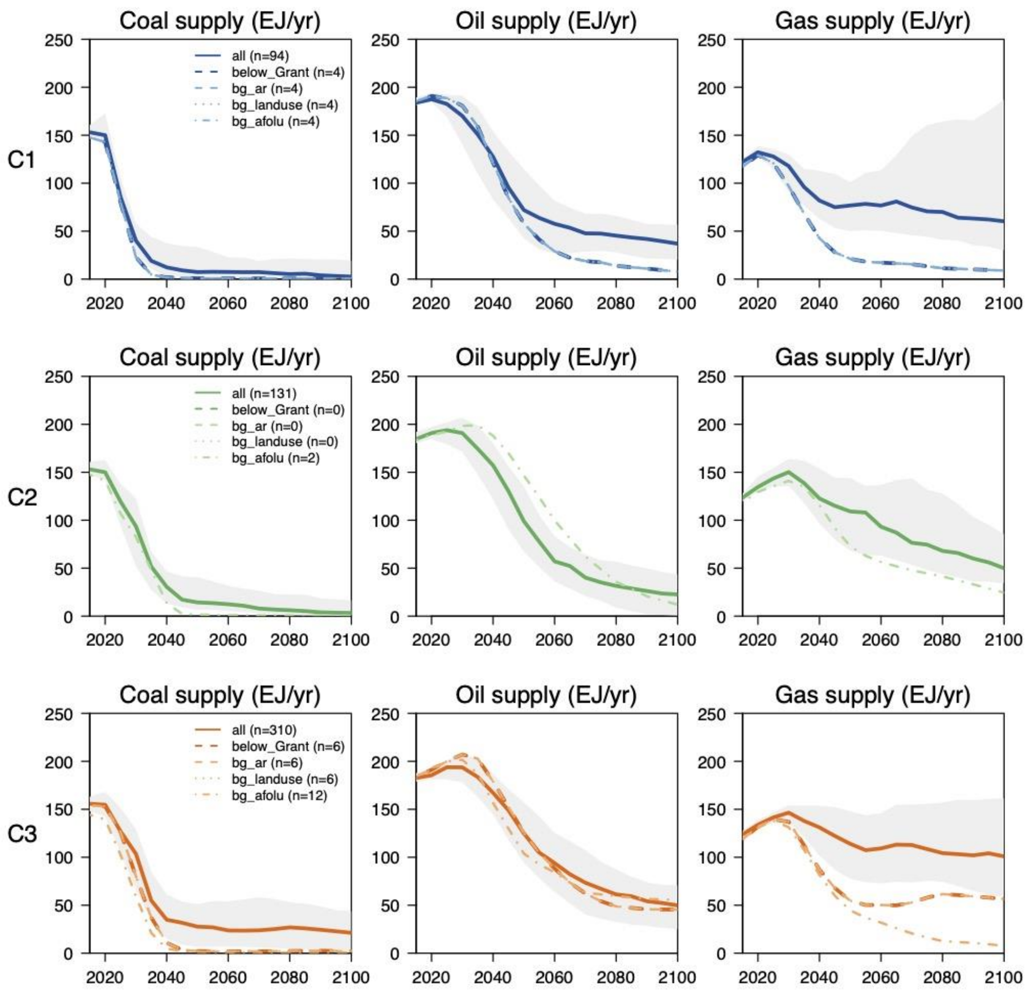

Supplementary Fig. 25. Sensitivity of the resulting median pathways of 2015-2100 coal, oil, and gas supply in the C1-C3 scenarios when using different model variables to constrain cumulative 2020-2100 afforestation (AR) to be equal to or less than 224 $GtCO_2$ (along with limiting BECCS and DACCS). In "bg_ar", the variable "Carbon Sequestration|Land Use|Afforestation" is used, which is reported by 42%-57% of the C1-C3 scenarios. In "bg_landuse", the variable "Carbon Sequestration|Land Use" is used, which has 62%-66% coverage. In "bg_afolu", the variable "Emissions|CO2|AFOLU" is used, which has 99%-100% coverage. In "below_Grant", which is the final method used, "Carbon Sequestration|Land Use" is applied in cases where "Carbon Sequestration|Land Use|Afforestation" is not reported.

Supplementary References

1. Riahi, K. *et al.* Cost and attainability of meeting stringent climate targets without overshoot. *Nat. Clim. Chang.* **11**, 1063–1069 (2021).
2. Joint Research Center. *GEM-E3 model documentation*. (Publications Office, 2013).
3. Grant, N., Gambhir, A., Mittal, S., Greig, C. & Köberle, A. C. Enhancing the realism of decarbonisation scenarios with practicable regional constraints on CO2 storage capacity. *International Journal of Greenhouse Gas Control* **120**, 103766 (2022).
4. Koelbl, B. S., van den Broek, M. A., Faaij, A. P. C. & van Vuuren, D. P. Uncertainty in Carbon Capture and Storage (CCS) deployment projections: a cross-model comparison exercise. *Climatic Change* **123**, 461–476 (2014).
5. Grubler, A. *et al.* A low energy demand scenario for meeting the 1.5°C target and sustainable development goals without negative emission technologies. *Nature Energy* **3**, 515–527 (2018).
6. Luderer, G. *et al.* Impact of declining renewable energy costs on electrification in low-emission scenarios. *Nat Energy* **7**, 32–42 (2022).
7. Soergel, B. *et al.* A sustainable development pathway for climate action within the UN 2030 Agenda. *Nat. Clim. Chang.* **11**, 656–664 (2021).
8. Riahi, K. *et al.* Chapter 3: Mitigation pathways compatible with long-term goals. in *Climate Change 2022: Mitigation of Climate Change. Contribution of Working Group III to the Sixth Assessment Report of the Intergovernmental Panel on Climate Change* (Cambridge University Press, 2022).
9. Guivarch, C. *et al.* Using large ensembles of climate change mitigation scenarios for robust insights. *Nat. Clim. Chang.* **12**, 428–435 (2022).
